# Hepcidin expression is associated with increased γ-secretase–mediated cleavage of neogenin in the liver

**DOI:** 10.1016/j.jbc.2024.107927

**Published:** 2024-10-24

**Authors:** Caroline A. Enns, Richard H. Zhang, Shall Jue, An-Sheng Zhang

**Affiliations:** Department of Cell, Developmental, and Cancer Biology, Oregon Health & Science University, Portland, Oregon, USA

**Keywords:** hepatocyte, iron, homeostasis, hormone, neogenin, hepcidin, α-secretase, γ-secretase

## Abstract

Neogenin (NEO1) is a ubiquitously expressed transmembrane protein. It interacts with hemojuvelin (HJV). Both NEO1 and HJV play pivotal roles in iron homeostasis by inducing hepcidin expression in the liver. Our previous studies demonstrated that this process depends on Neo1–Hjv interaction and showed that the Hjv-mediated hepcidin expression is correlated with the accumulation of a truncated and membrane-associated form of Neo1. In this study, we tested whether hepcidin expression is induced by increased γ-secretase–mediated cleavage of Neo1 in the liver. We found that Neo1 underwent cleavage of its ectodomain and intracellular domains by α- and γ-secretases, respectively, in hepatoma cells. Our *in vitro* studies suggest that γ-secretase is responsible for cleavage and release of the cytoplasmic domain of Neo1 in the Hjv–Neo1 complex. This process was enhanced by the inhibition of α-secretase proteolysis and by co-expression with the Neo1-binding partner, Alk3. Further *in vivo* studies indicated that Neo1 induction of hepcidin expression required γ-secretase cleavage. Interestingly, neither predicted form of γ-secretase–cleaved Neo1 was able to induce hepcidin when separately expressed in hepatocyte-specific *Neo1* KO mice. These results imply that the function of Neo1 requires a *de novo* γ-secretase proteolysis. Additional studies revealed that in addition to the Hjv-binding domains, the function of Neo1 also required its C-terminal intracellular domain and the N-terminal immunoglobulin-like domains that are involved in Neo1 binding to Alk3. Together, our data support the idea that Neo1 induction of hepcidin is initiated as a full-length form and requires a *de novo* γ-secretase cleavage of Neo1’s cytoplasmic domain.

Neogenin (NEO1) is a multifunctional receptor that binds members of the repulsive guidance molecules, RGMa, RGMb, and hemojuvelin (HJV, also called RGMc). It consists of four immunoglobulin-like (Ig) domains, six fibronectin type III-like (FNIII) repeats, a single transmembrane domain (TMD), and an intracellular domain (ICD) (Fig. 1*A*). NEO1 interacts with the RGMs through its FNIII 5 to 6 domains (1, 2, 3, 4). RGMa and RGMb are mainly restricted to the developing nervous system and are necessary for neural development (5). Like the other RGMs, HJV is a glycosylphosphatidylinositol-linked membrane protein. In contrast to the other RGMs, HJV is predominantly expressed in hepatocytes, skeletal muscle cells, and cardiomyocytes. Mutations in the *HJV* gene diminish hepatic hepcidin expression leading to juvenile hemochromatosis, a particularly severe form of iron overload disorder in humans (6).

**Figure 1:**
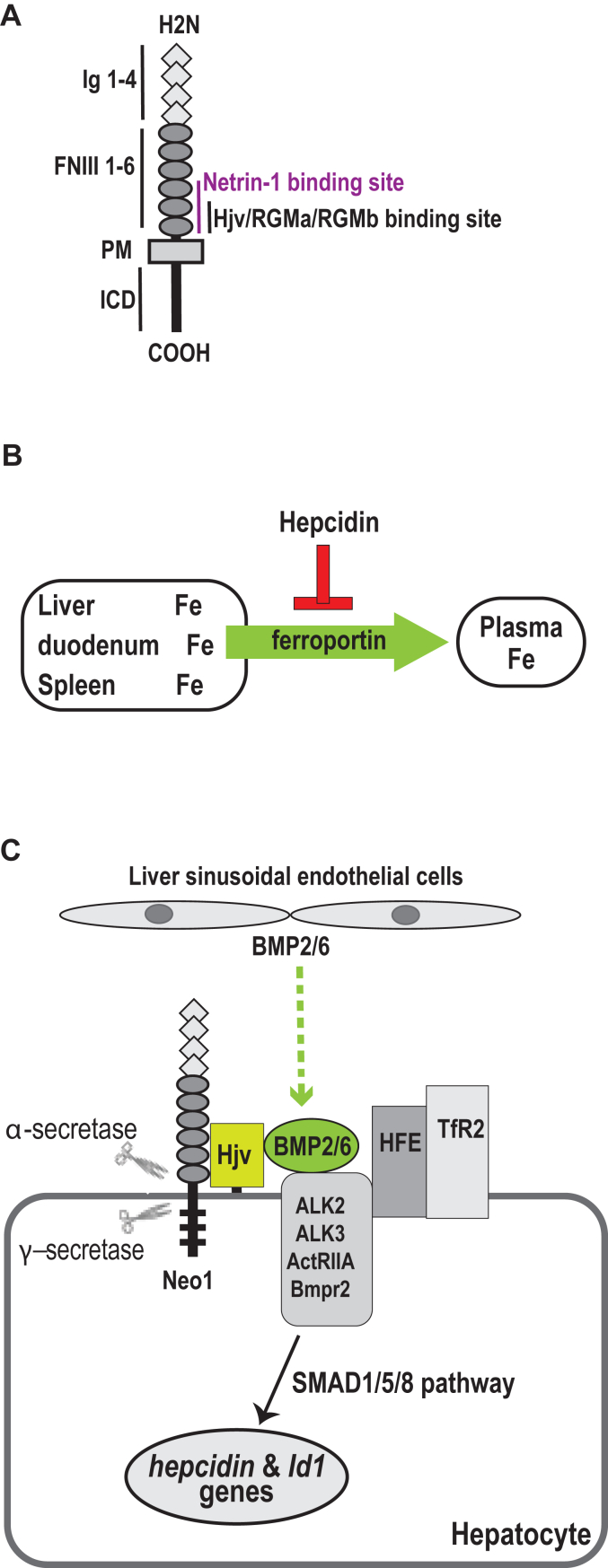
**Neogenin and other key players in the induction of hepcidin expression in the liver.***A*, diagram of Neo1. *B*, hepcidin inhibits iron efflux from duodenum, spleen, and the liver into the circulation by blocking the plasma-membrane iron exporter, ferroportin. *C*, diagram of the key components that are involved in the induction of hepcidin expression in the liver. FNIII, fibronectin III; HFE, the hemochromatosis protein; ICD, intracellular domain; Id1, the inhibitor of differentiation-1 gene; Ig, immunoglobulin-like domain; PM, plasma membrane.

Hepcidin is an iron regulatory hormone that is secreted mainly by hepatocytes. It downregulates iron efflux from duodenal epithelial cells, macrophages, and hepatocytes into the circulation by directly occluding the plasma membrane iron exporter, ferroportin, as well as by inducing its internalization and degradation (7, 8) (Fig. 1*B*). Murine studies demonstrate that hepatic Hjv is critical for normal hepcidin expression and iron homeostasis (9, 10, 11, 12). Hepatic hepcidin expression is induced *via* the BMP-signaling pathway by utilizing a selective set of BMP ligands (BMP2 and BMP6), BMP receptors (ALK2, ALK3, ActRIIA, and Bmpr2), and cytoplasmic SMADs (SMAD1/5/8) (13, 14, 15, 16, 17, 18, 19, 20, 21, 22, 23, 24) (Fig. 1*C*). Hepatocyte HJV acts as a coreceptor for BMP6 and uses two type-I BMP receptors, ALK2 and ALK3, to robustly stimulate hepcidin expression *via* the BMP-signaling pathway (14, 16, 22, 23, 25). Additionally, a normal range of hepcidin expression also requires the plasma proteins, the hemochromatosis protein, and transferrin receptor-2 (TfR2) (26). Structural studies show that HJV can simultaneously bind NEO1 and ALK3 (or BMP ligand) through its C-terminal and N-terminal portions, respectively (3, 4). We also found that in the absence of HJV, NEO1 can interact with ALK3 in a manner that requires the NEO1-Ig domains (27). ALK3 is a pivotal type-I BMP receptor for hepcidin expression (16). Thus, NEO1 has the potential to form a complex with the key hepcidin-inducing components, including HJV, ALK3, and BMPs.

Recent studies demonstrate that hepatic Neo1 plays a pivotal role in hepcidin expression and in iron homeostasis (28). Ablation of hepatic *Neo1* decreases hepcidin expression and causes iron overload in mice (28). Mechanistic studies reveal that the function of hepatic Neo1 relies on its interaction with Hjv (27, 28). Consistently, the most common juvenile hemochromatosis-causing mutation in HJV, G320V, lowers its interaction with NEO1 (1, 3). Global *Neo1* mutant mice also have reduced hepcidin expression and severe iron overload that is indistinguishable from *Hjv*^*−/−*^ mice, except that the former displays severe developmental defects and perinatal death (29, 30, 31). The underlying mechanism by which hepatic Neo1 induces hepcidin remains unknown.

The perinatal death of global *Neo1* mutant mice implies that Neo1 is involved in other pathways. NEO1 is also a receptor for netrins, including netrin-1, 2, and 3. It binds netrin-1 through its FNIII 4 to 6 domains (Fig. 1*A*) and can form a ternary NEO1–Netrin-1–RGM complex (32). Netrin-1 is a secreted cytokine that is expressed primarily by the neurons. It has low levels of expression in other tissues including the liver (33). Netrin-1 plays a central role in cell adhesion, cell migration, proliferation, cell survival, and inflammation in neuronal and non-neuronal tissue (33, 34, 35). Whether Netrin-1 is involved in iron homeostasis is not known.

A host of observations suggest that hepatic Neo1 induction of hepcidin expression requires Hjv-facilitated cleavage of full-length Neo1. NEO1 can be cleaved by α- and γ-secretases (36, 37, 38, 39). The α-secretase cleaves NEO1 extracellularly at a site adjacent to the transmembrane domain, whereas γ-secretase cleaves NEO1 within the transmembrane domain and releases its complete ICD into the cytosol (Figs. 1*C* and 2*A*) (38). The precise cleavage sequences are not known. Emerging evidence support that the function of NEO1 in neurons requires γ-secretase proteolysis of its ICD but does not need α-secretase cleavage. In HEK293 and MFC7 cells, the γ-secretase–cleaved NEO1-ICD translocates to the nucleus to upregulate the transcription of target genes in the presence of RGMa (38). In zebrafish, RGMa promotes γ-secretase proteolysis of Neo1 and results in a transient nuclear localization of Neo1-ICD. Overexpression of Neo1-ICD is able to partially rescue the Neo1/RGMa knockdown embryos (39). In mice, the seizure-induced hippocampal necroptosis is associated with the increased generation of truncated Neo1 by γ-secretase proteolysis (40). In contrast, the α-secretase cleavage of Neo1 ectodomain is reported to terminate the RGMa-induced Neo1 signaling in neurons (36). Interestingly, our previous studies show that the Hjv-mediated hepcidin expression is also associated with an accumulation of truncated Neo1 (Neo1-ECT/TMD; Fig. 2*B*) in the liver (28). This truncated form of hepatic Neo1 is membrane-associated, and it is derived from the cleavage in its C terminus by an undefined protease (28).

**Figure 2:**
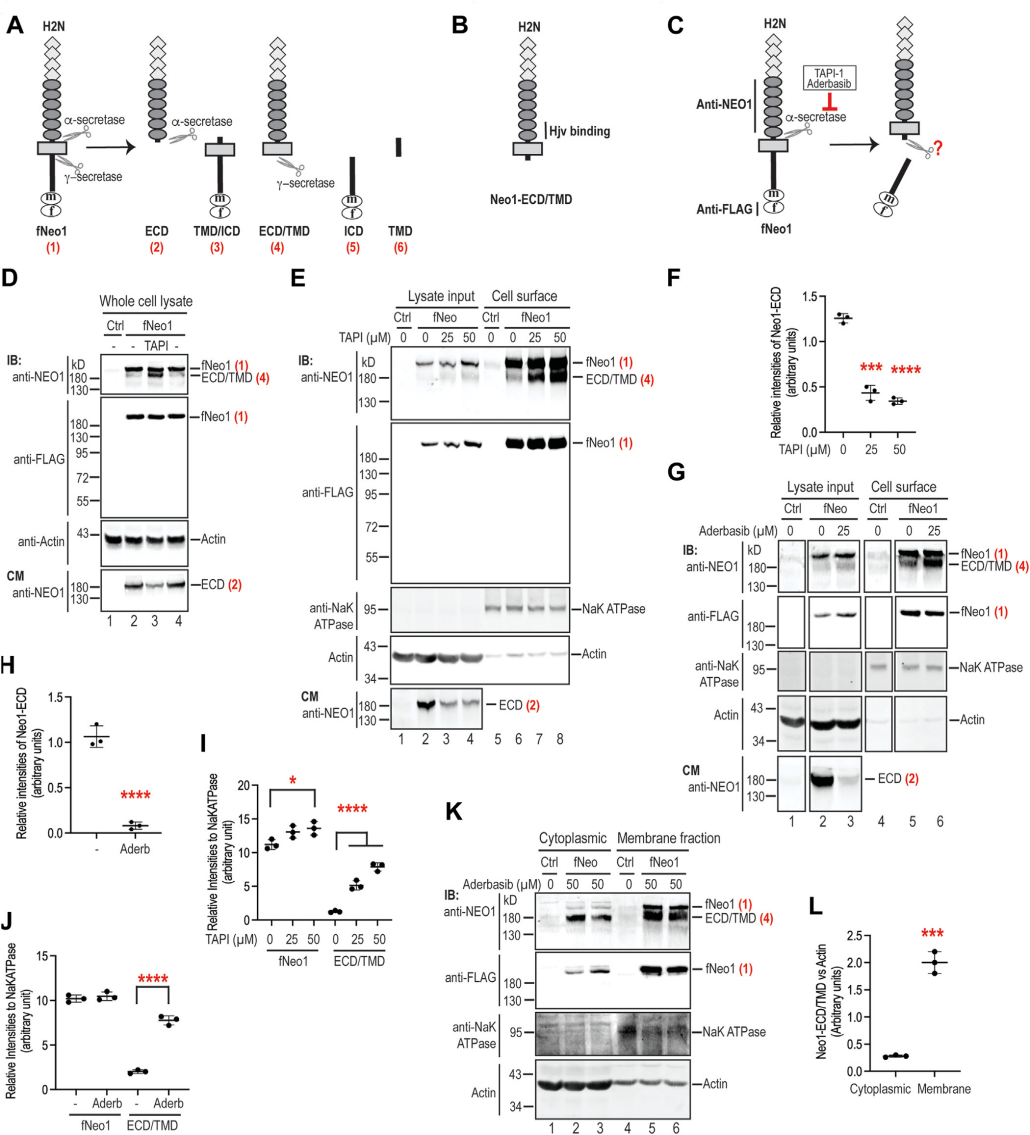
**Inhibition of α-secretase proteolysis of Neo1 ectodomain increases truncated Neo1-ECD/TMD in Hep3B cells.***A*, diagram of the full-length fNeo1 and the predicted cleavage products by α- and γ-secretases. The numbers in *red* are given for the convenience to locate the corresponding bands in the Western blot images. *B*, predicted form of Neo1 that is increased by Hjv in the liver. *C*, α-secretase inhibitors and the antibodies used for studies. *D*, incubation with TAPI-1 inhibits the ECD shedding of fNeo1 in Hep3B cells. Cells were incubated with 20 μM TAPI-1 for ∼16 h. The cell lysate and a fraction of concentrated conditioned medium (CM) were subjected to SDS-PAGE (11%) and immunodetection (IB). *E*, inhibition of α-secretase with TAPI-1 increases truncated fNeo1-ECD/TMD on cell surface. After fNeo1-transfected Hep3B cells were incubated in the presence of 25 and 50 μM TAPI-1 for ∼16 h, cell surface proteins were biotinylated. The eluted cell surface proteins, ∼10% of input lysate, and a fraction of concentrated CM were subjected to SDS-PAGE (9%) and immunodetection. *F*, quantification of Neo1-ECD bands in (*E*) (n = 3). *G*, inhibition of α-secretase with Aderbasib increases truncated fNeo1-ECD/TMD on cell surface. Experiments were performed as described above in (*E*). Each panel was cropped from the same image. *H*, quantification of Neo1-ECD bands in (*G*) (n = 3). *I*, quantification of cell surface fNeo1 and fNeo1-ECD/TMD bands in (*E*) (n = 3). *J*, quantification of cell surface fNeo1 and fNeo1-ECD/TMD bands in (*G*) (n = 3). *K*, Western blot analysis of fNeo1, fNeo1-ECD/TMD, Na^+^K^+^ATPase, and β-actin in cytoplasmic and membrane fractions from fNeo1-transfected Hep3B cells (∼5 × 10^6^) after incubation with Aderbasib for ∼16 h. *L*, quantification of fNeo1-ECD/TMD bands in cytoplasmic and membrane fractions in (*K*) (n = 3). All experiments were repeated at least three times with consistent results. All quantification data shown are means ± SD. ∗, *p* < 0.05; ∗∗∗, *p* < 0.001; ∗∗∗∗, *p* < 0.0001. ECD, ectodomain; f, FLAG tag; m, MYC tag; TMD, transmembrane domain.

In this study, we tested the hypothesis that hepatic Neo1 induction of hepcidin expression is associated with increased γ-secretase–mediated cleavage of Neo1. Our *in vitro* studies indicate that the Hjv-mediated increase of Neo1 in the liver (28) is derived from γ-secretase proteolysis of Neo1 intracellular domain. Our *in vivo* data demonstrate that hepatic Neo1 induction of hepcidin requires not only its Hjv-binding domain but also other domains and suggest that this process needs a γ-secretase proteolysis of full-length Neo1.

## Results

### Inhibition of α-secretase proteolysis of Neo1 ectodomain in Hep3B cells recapitulates the Hjv-mediated increases of truncated Neo1 in the liver

We used Hep3B cells as a model to determine whether the Hjv-mediated accumulation of hepatic Neo1 that lacks a portion of its C-terminal intracellular domain (Fig. 2*B*) (28) resulted from the inhibition of α-secretase proteolysis of its ectodomain. Hep3B cells are a human hepatoma cell line that endogenously expresses both α- and γ-secretases and possess many characteristics of hepatocytes (28, 41, 42). Although it expresses more than 100-fold lower levels of HJV mRNA than those in the liver (28), it is an ideal model to study the processing of iron-regulatory proteins. To immunodetect different cleaved forms, a murine Neo1 construct with a C-terminal FLAG/MYC epitope (fNeo1; Fig. 2*A*) was used for transfection. Addition of a C-terminal FLAG/MYC epitope did not impact the induction of hepcidin expression in mice (28). Since the precise cleavage sequence by α-secretase in Neo1 is unknown, specific α-secretase inhibitors were employed (Fig. 2*C*). As expected, full-length fNeo1 migrating at ∼230 kD was detected in cell extracts by both anti-NEO1 and anti-FLAG antibodies (Fig. 2*D*; panels 1/2; lanes 2/4). Consistent with previous studies (28, 38), a putative α-secretase–cleaved fNeo1 ectodomain (fNeo-ECD) migrating at ∼170 kD was observed in the conditioned medium by an anti-NEO1 antibody. Its levels were markedly decreased by incubation with TAPI-1, an α-secretase inhibitor (Fig. 2*D*, lowest panel; lane 3; Fig. 2*E*, lowest panel; lanes 3/4; Fig. 2*F*) and almost completely abolished when incubated with Aderbasib (INCB7839), a potent α-secretase inhibitor (Fig. 2*G*, lowest panel; lane 3; Fig. 2*H*). The used concentrations were based on earlier studies of TAPI-1 and INCB3619, an analogous inhibitor to Aderbasib (39, 43). Based on the molecular weight of shed fNeo1-ECD in the conditioned medium and the localization of the protease domain of α-secretase, α-secretase is predicted to cleave Neo1 extracellularly at a site adjacent to the transmembrane domain in hepatoma cells.

Intriguingly, inhibition of α-secretase cleavage resulted in a significant increase of truncated fNeo1 at ∼180 kD in cell extracts, which migrated right beneath the full-length fNeo1 in SDS-PAGE under the reducing and denaturing conditions (Fig. 2*D*; panel 1; lane 3). This form of fNeo1 was immunodetected by anti-NEO1 antibody, but not anti-FLAG antibody (Fig. 2*D*; panels half; lane 3), indicating that it was derived from a cleavage within its C-terminal region. To determine whether it was the α-secretase–cleaved fNeo1-ECD remaining within the cells, we performed cell surface biotinylation analysis. As shown in Fig. 2*E* (panels half; lanes 6–8), this truncated fNeo1 was mainly detected on the cell surface, similarly to full-length fNeo1 and NaK-ATPase, a plasma membrane marker. As a negative control, a negligible amount of β-actin was found in the eluates of biotinylated cell surface proteins (Fig. 2*E*; panel 4; lanes 5–8). These results suggest that this truncated fNeo1 is associated with the plasma membrane and that it is analogous to Hjv-induced Neo1-ECD/TMD in the liver (28). Notably, the cell surface fNeo1-ECD/TMD levels were significantly elevated after incubation with the α-secretase inhibitors, TAPI-1 (Fig. 2*E*; panel 1, lanes 6–8; Fig. 2*I*) and Aderbasib (Fig. 2*G*; panel 1; lanes 5–6; Fig. 2*J*). The full-length fNeo1 was also increased modestly by TAPI-1 (Fig. 2. *E* and *I*). These results suggest that the increased fNeo1-ECD/TMD by α-secretase inhibitors is derived from the cleavage of full-length fNeo1 within its intracellular domain.

To further ascertain that this truncated fNeo1-ECD/TMD is membrane-associated, we performed subcellular fractionation of the fNeo1-expressing Hep3B cells after incubation with Aderbasib. In agreement with the biotinylation analysis of cell surface proteins as described above (Fig. 2*G*), Western blot analysis revealed that the majority of truncated fNeo1-ECD/TMD was detected in the membrane fractions by anti-NEO1 antibody, but not by anti-FLAG antibody that recognizes the C-terminal intracellular domain (Fig. 2*K*; panels 1/2; lanes 2/3 vs 5/6; Fig. 2*L*). As positive controls, both full-length fNeo1 and NaK-ATPase was predominantly found in the membrane fractions (Fig. 2*K*; panels 1–3). As a negative control, β-actin were mainly detected in the cytoplasmic fractions (Fig. 2*K*, lowest panel). Together, these observations indicate that this truncated fNeo1-ECD/TMD is membrane-associated and that it is derived from a cleavage within the intracellular domain.

### The truncated Neo1-ECD/TMD is likely derived from a direct γ-secretase proteolysis of full-length Neo1

**Figure 3:**
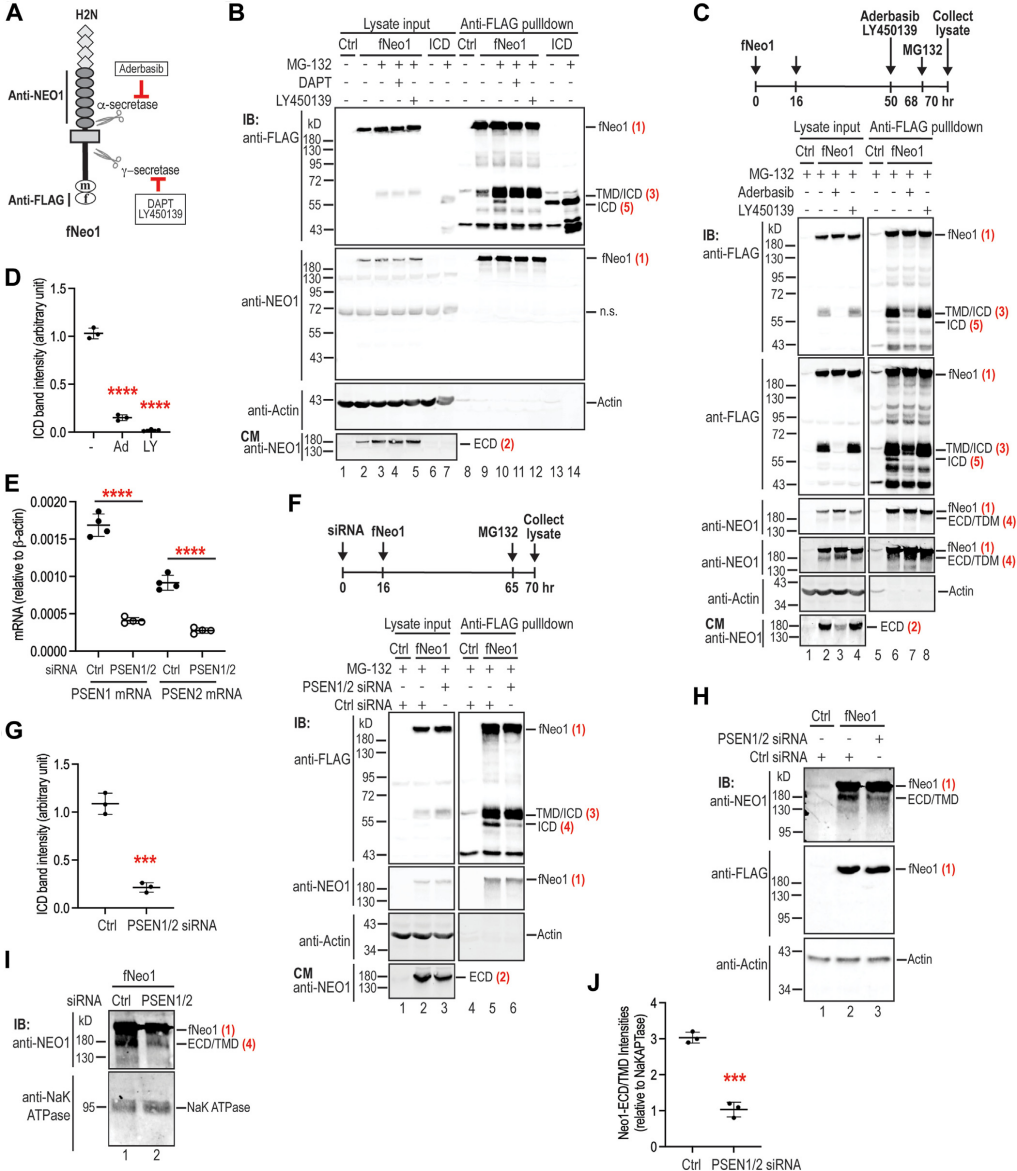
**Knockdown of γ-secretase diminishes the generation of membrane-associated fNeo1-ECD/TMD in Hep3B cells.***A*, α- and γ-secretase inhibitors used for studies. *B*, incubation with DAPT and LY450139 abolishes the release of Neo1-ICD. Hep3B cells were transiently transfected with pCMV6-fNeo1 or fNeo1-ICD construct. Cells were treated with 10 μM MG-132 and 10 μM DAPT or 10 μM LY450139 for about 7 h. About 90% of the cell lysate was subjected to pull-down using anti-FLAG affinity gel. The eluted proteins, ∼10% of input lysate, and a fraction of concentrated CM were subjected to SDS-PAGE (11%) and immunodetection. n.s., nonspecific band. *C*, inhibition of α-secretase by Aderbasib (25 μM) reduces γ-secretase cleavage of transfected fNeo1 in Hep3B cells. Incubation with LY450139 (10 μM) was included as a positive control. Experiments were performed as described above in (*B*). Each panel was cropped from the same image. *D*, quantification of fNeo1-ICD bands in (*C*) (panel-2, lane 6–8; n = 3). *E*, quantitative reverse transcription-PCR (qRT-PCR) analysis of PSEN1 and PSEN2 mRNA levels in Hep3B cells after transfection with PSEN1, PSEN2, or control (Ctrl) siRNA. Results are expressed as the amount relative to that of β-Actin (n = 4). Data shown are means ± SD. *F*, knockdown of PSEN1 and PSEN2 (PSEN) by specific siRNAs reduces the generation of fNeo1-ICD in fNeo1-transfected Hep3B cells. Each panel was cropped from the same image. *G*, quantification of fNeo1-ICD bands in (F) (panel-1, lane 5–6; n = 3). *H*/*I*, knockdown of PSEN1 and PSEN2 (PSEN) by specific siRNAs reduces the levels of fNeo1-ECD/TMD in cell lysate (*H*) and on cell surface (*I*) of fNeo1-transfected Hep3B cells. *J*, quantification of fNeo1-ECD/TMD bands in (*I*) (n = 3). All experiments were repeated at least three times with consistent results. All quantification data shown are means ± SD. ∗∗∗, *p* < 0.001; ∗∗∗∗, *p* < 0.0001.

To determine whether the Hjv-increased Neo1-ECD/TMD in the liver (28) is derived from γ-secretase cleavage of fNeo1 intracellular domain (fNeo1-ICD), we first tested if fNeo1 undergoes γ-secretase proteolysis in Hep3B cells by using specific inhibitors (Fig. 3*A*). The γ-secretase–cleaved fNeo1-ICD was evaluated after incubation of transfected Hep3B cells with the proteosome inhibitor MG132 to inhibit the turnover of cleaved fNeo1-ICD. The cleaved NEO1-ICD undergoes a rapid proteasome degradation in breast cancer cell lines and neuronal extracts (38). We concentrated all FLAG-containing forms of fNeo1 from cell extracts by using the anti-FLAG bead pulldown for analysis. In agreement with the previous study (38), a distinct fNeo1 band migrating at ∼55 kD was immunodetected in the pulldown eluate by an anti-FLAG antibody (Fig. 3*B*; panel 1; lane 10), which migrated similarly to the transfected fNeo1-ICD (Fig. 3*B*; panel 1; lanes 13/14). This fNeo1-ICD band was abolished by incubation with γ-secretase inhibitors, DAPT or LY450139 (Fig. 3*B*; panel 1; lanes 11/12). The concentrations used for these inhibitors were based on the earlier *in vitro* studies (39, 44, 45). These results indicate that fNeo1 is indeed cleaved by γ-secretase in hepatoma cells. In the presence of γ-secretase inhibitors, the unchanged fNeo1-ECD levels in the conditioned medium (Fig. 3*B*; lowest panel, lanes 3–5; Fig. S1) suggest that blockage of γ-secretase cleavage does not impact α-secretase proteolysis of fNeo1. The band migrating at ∼60 kD right above the fNeo1-ICD recognized by the anti-FLAG antibody (Fig. 3*B*; panel 1; lane 10) is likely the α-secretase–cleaved fNeo1-TMD/ICD (Fig. 2*A*), because it was detected on the cell surface after inhibition of γ-secretase (Fig. S1). The marked increases of this cell surface fNeo1-TMD/ICD by γ-secretase inhibitors (Fig. S1) suggest that γ-secretase is the predominant protease to cleave Neo1 intracellular domain in hepatoma cells.

γ-Secretase cleaves numerous type-I transmembrane proteins (46). Typically, γ-secretase–mediated intramembrane proteolysis is preceded by ectodomain shedding of the substrates by α- or β-secretase (47). To test whether this sequential cleavage process applies to Neo1, we examined the impact of α-secretase inhibition by Aderbasib on γ-secretase cleavage of transfected fNeo1 in Hep3B cells. Incubation with the γ-secretase inhibitor, LY450139, was included as a positive control. As expected, treatment with Aderbasib markedly reduced the levels of shed Neo1-ECD in the conditioned medium (Fig. 3*C*; lowest panel; lane 3). The parallel decreases of fNeo1-TMD/ICD levels in both the lysate input and anti-FLAG pulldown eluate (Fig. 3*C*; top panel; lanes 3 & 7) indicate that it is the membrane-associated portion of α-secretase–cleaved fNeo1. Interestingly, the fNeo1-ICD level was markedly reduced by Aderbasib inhibition of α-secretase activity (Fig. 3*C*; panels half; lane 7; Fig. 3*D*). As a positive control, incubation with LY450139 completely abolished the generation of fNeo1-ICD (Fig. 3*C*; panel 2; lane 8; Fig. 3*D*) with no evident effect on the levels of α-secretase–cleaved Neo1-ECD (Fig. 3*C*; lowest panel; lane 4) and fNeo1-TMD/ICD (Fig. 3*C*; panel 1; lanes 4 & 8). These observations indicate that the γ-secretase–mediated proteolysis of Neo1, to a large extent, depends on the ectodomain shedding by α-secretase, but not *vice versa*, in Hep3B cells.

γ-Secretase is a multiprotein complex that contains the redundant catalytic (Presenilin1 or 2; PSEN1, PSEN2) and regulatory (Aph-1a or -1b) subunits as well as the unique targeting (Nicastrin) and enhancer (Pen2) components (46, 47). Quantitative reverse transcription-PCR (qRT-PCR) analysis revealed that Hep3B cells express both PSEN1 and PSEN2 mRNA (Fig. 3*E*). To ascertain whether the detected fNeo1-ECD/TMD is derived from a direct γ-secretase proteolysis, we knocked down both PSEN1 and PSEN2 in Hep3B cells by using specific siRNAs, followed by a transient transfection of fNeo1. As predicted, a simultaneous depletion of both PSEN1 and PSEN2 mRNA by ∼70% (Fig. 3*E*) was able to significantly reduce fNeo1-ICD levels in the anti-FLAG pulldown eluates when compared with the control siRNA (Fig. 3*F*; top panel; lanes 5/6; Fig. 3*G*). These data validated that fNeo1 indeed undergoes γ-secretase–mediated proteolysis in hepatoma cells. Interestingly, the simultaneous PSEN1 and PSEN2 knockdown resulted in a significant decrease of fNeo1-ECD/TMD levels both in the whole cell extracts (Fig. 3*H*; top panel; lanes 2/3) and in the eluates of biotinylated cell surface proteins (Fig. 3*I*; top panel; Fig. 3*J*). These results suggest that the membrane-associated fNeo1-ECD/TMD is derived from a direct γ-secretase proteolysis. In conjunction with the studies using γ-secretase inhibitors above (Fig. 3*J*), these observations imply that in the absence of Neo1-binding partners, only a fraction of transfected fNeo1 undergoes this unique processing pathway.

### Alk3 facilitates the generation of γ-secretase–cleaved Neo1-ECD/TMD, and Hjv prevents netrin-1–induced Neo1 degradation in Hep3B cells

**Figure 4:**
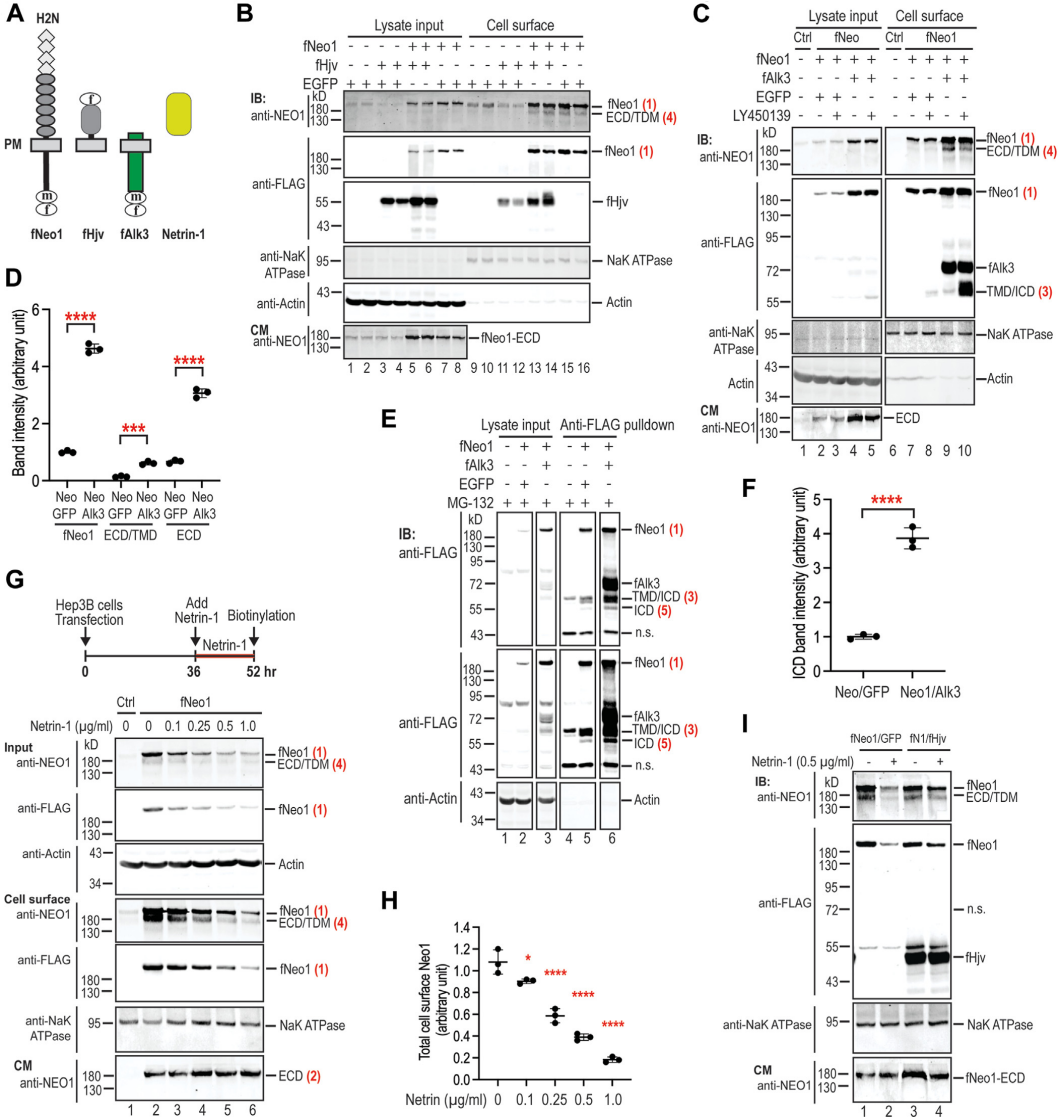
**Hjv prevents Netrin-1–induced Neo1 degradation, and Alk3 facilitates the generation of γ-secretase–cleaved Neo1-ECD/TMD in Hep3B cells.***A*, diagram of fNeo1, mouse Hjv with an N-terminal 3xFLAG epitope (fHjv), mouse Alk3 with an N-terminal FLAG/MYC epitope (fAlk3), and mouse Netrin-1. *B*, co-expression with fHjv does not inhibit α-secretase cleavage of fNeo1. At 56 h after transfection, cell surface proteins were biotinylated. The eluted cell surface proteins, ∼10% of input lysate, and a fraction of concentrated CM were subjected to SDS-PAGE and immunodetection (IB). *C*, co-expression with fAlk3 increases fNeo1, fNeo1-ECD/TMD, and fNeo1-ECD. Experiments were performed as described above in (*B*) except that LY450139 (10 μM) was used to inhibit γ-secretase proteolysis. Each panel was cropped from the same image. *D*, quantification of cell surface fNeo1 and fNeo1-ECD/TMD bands in (*C*) (panel-1, lane 7/9), as well as fNeo1-ECD bands in (*C*) (lowest panel, lane 2/4) (n = 3). *E*, co-expression with fAlk3 increases γ-secretase–cleaved fNeo1-ICD. Experiments were performed as described in the legend to Fig. 3*B*. Each panel was cropped from the same image. Two sets of images with different exposure for anti-FLAG antibody were presented. *F*, quantification of fNeo1-ICD bands in (*E*) (panel-2; lane 5/6) (n = 3). *G*, incubation with Netrin-1 decreases fNeo1 levels in Hep3B cells. fNeo1-transfected Hep3B cells were incubated with mouse Netrin-1 at 0, 0.1, 0.25, 0.5, or 1.0 μg/ml for ∼16 h. Cell surface proteins were subjected to biotinylation and immunodetection. *H*, quantification of total cell surface fNeo1 in (*G*) (panel-4) (n = 3). *I*, co-expression of Hjv and Neo1 prevents Netrin-1–mediated degradation of Neo1. Cells were transfected as described above in (*B*) and incubated with mouse Netrin-1 at 0 and 0.5 μg/ml for ∼16 h, followed by biotinylation and immunodetection. All experiments were repeated at least three times with consistent results. All quantification data shown are means ± SD. ∗, *p* < 0.05; ∗∗∗, *p* < 0.001; ∗∗∗∗, *p* < 0.0001.

NEO1 binds HJV, ALK3 (a type-I transmembrane receptor), and netrin-1 (a secreted cytokine) (3, 4, 27, 32) (Fig. 4*A*). Earlier studies suggest that the Neo1/Hjv-mediated hepcidin expression is accomplished by forming a complex with other key hepcidin-inducing components in the liver (3, 4, 14, 16, 22, 23, 25) (Fig. 1*C*). To seek insight into the mechanism by which Hjv increases Neo1 in the liver (28), we tested the impact of these Neo1-binding partners on α- and γ-secretase proteolysis of Neo1 in Hep3B cells. Hjv binds to the predicted region in Neo1 where the α-secretase proteolysis occurs (Fig. 1*A*). The effects of Hjv were examined by the co-expression of mouse fNeo1 and mouse Hjv with a N-terminal 3xFLAG epitope (fHjv; Fig. 4*A*). Addition of an N-terminal 3xFLAG epitope did not affect Hjv induction of hepcidin expression in mice (27, 28). Co-expression with EGFP was used as a control to balance the amount of protein synthesis in cells. In contrast to our prediction, co-expression of fHjv with fNeo1 was unable to significantly increase fNeo1 levels either in the cell extracts or on the cell surface when compared with cells that expressed fNeo1/EGFP (Fig. 4*B*; panel 1, lanes 5–8 & 13–16). Rather, we detected a mild increase in α-secretase proteolysis of fNeo1 by fHjv as manifested by a modest increase in the levels of shed fNeo1-ECD in the conditioned medium (Fig. 4*B*; lowest panel, lanes 5/6 vs 7/8; Fig. S2*A*). These observations imply that the association with a single binding partner at the α-secretase cleavage site is insufficient to block α-secretase proteolysis of Neo1. No evident effect on γ-secretase cleavage of fNeo1 by fHjv were observed (Fig. S2*A*). Since Hep3B cells are a relatively undifferentiated hepatoma cell line and do not express many of the genes that are involved in iron homeostasis, these observations indicate that expression of Hjv alone is insufficient to increase Neo1 and suggest that other components are involved in the Hjv-mediated increase in Neo1 in the liver.

The effects of Alk3 were evaluated next by employing a similar approach as described for fHjv. Alk3 contains a short ectodomain, a single-span transmembrane domain, and a large cytoplasmic domain, and it is an essential type-I BMP receptor for hepcidin expression in the liver (16). Interestingly, in contrast to fHjv, co-expression with murine Alk3 containing a C-terminal FLAG/MYC epitope (fAlk3) resulted in a marked increase of both full-length fNeo1 and fNeo1-ECD/TMD on cell surface by biotinylation (Fig. 4*C*; top panel, lanes 7/9), as well as a parallel increase in α-secretase–cleaved fNeo1-ECD in the conditioned medium (Fig. 4*C*; lowest panel; lanes two-fourths; Fig. 4*D*). Addition of a C-terminal FLAG/MYC epitope did not affect Alk3 induction of hepcidin expression in mice (unpublished observations). Anti-FLAG bead pulldown analysis revealed that expression of fAlk3 also led to a significant elevation of γ-secretase–cleaved fNeo1-ICD and α-secretase–cleaved fNeo1-TMD/ICD (Fig. 4*E*; panel 1/2; lanes 5/6; Fig. 4*F*). These results indicate that the Neo1/Alk3 association stabilizes Neo1, which leads to a proportional increase in both α- and γ-secretase proteolysis of Neo1. The significant increase of cell surface fNeo1-ECD/TMD by fAlk3 was likely derived from the direct γ-secretase proteolysis of fNeo1, because its level was reduced by incubation with the γ-secretase inhibitor, LY450139 (Fig. 4*C*; top panel; lane 9/10). Consistently, the decrease of cell surface fNeo1-ECD/TMD by LY450139 was associated with an increase of α-secretase–cleaved fNeo1-TMD/ICD on the cell surface (Fig. 4*C*; panels 1/2, lanes 9/10). Together, these observations suggest that Alk3 facilitates the generation of γ-secretase–cleaved fNeo1-ECD/TMD by the stabilization of full-length Neo1 in Hep3B cells. In comparison with fHjv, the robust effects of fAlk3 imply that the transmembrane and intracellular domains of Neo1-binding partner play an important role in the regulation of Neo1 stability and proteolysis.

To seek insight into the potential roles of the transmembrane and intracellular domains of the Neo1-binding partner, we examined the effects of netrin-1 on α- and γ-secretase proteolysis of fNeo1. Netrin-1 is a secreted cytokine that binds to the FNIII 4 to 6 domains of Neo1 (32), similarly to Hjv (Fig. 1*A*). The effects of netrin-1 were assessed by the addition of recombinant mouse netrin-1 (#1109-N1; R&D Systems) to the culture medium of fNeo1-transfected Hep3B cells. As shown in Fig. 4*G*, incubation with netrin-1 for ∼16 h resulted in a marked decrease in both cellular (input) and cell surface fNeo1 in a dose-dependent manner (Fig. 4*H*). Similarly to fNeo1/fHjv co-expression, we detected a mild increase in α-secretase–cleaved fNeo1-ECD in the conditioned medium by netrin-1 (Fig. 4*G*; lowest panel, lanes 2–6) with no evident effect on γ-secretase proteolysis of fNeo1 (Fig. S2*B*). This netrin-1–mediated reduction of fNeo1 could be largely prevented by incubation with MG132, an inhibitor of proteosome-mediated protein degradation (Fig. S2*B*). Since netrin-1 is not tethered to the plasma membrane, these results suggest that the binding of a membrane-untethered partner results in a rapid proteasomal degradation of Neo1.

Earlier *in vitro* studies show that both HJV and netrin-1 can simultaneously bind to NEO1 (32). We next examined the impact of a forced formation of the ternary fNeo1–Hjv–netrin-1 complex on the proteolysis of fNeo1 in Hep3B cells. Interestingly, when compared with the fNeo1/EGFP-expressing controls, co-expression of fNeo1/fHjv was able to largely prevent the Netrin-1-mediated reduction of cell surface fNeo1 (Fig. 4*I*; panels-1/2, lanes 2/4). These results suggest that the Neo1/Hjv association can prevent netrin-1–mediated Neo1 degradation. Taken together, the above *in vitro* studies imply that the Hjv-increased Neo1-ECD/TMD in the liver (28) is derived from γ-secretase proteolysis and that this process is facilitated by the association of Neo1 with its membrane-associated binding partners to form a complex in hepatocytes.

### Inhibition of γ-secretase activity reduces hepatic hepcidin expression in mice

To determine the roles of γ-secretase proteolysis of Neo1 in hepcidin expression, we first examined the effects of γ-secretase inhibitors, LY450139 and DAPT, in Hep3B and HepG2 cells with or without increased fNeo1 or NEO1 expression. Both hepatoma cell lines endogenously express low levels of NEO1 and hepcidin (Figs. S3 and S4). Incubation with neither of these inhibitors was able to significantly alter BMP6 induction of *hepcidin* or *ID1* expression in these cells (Fig. S4). *ID1* (*Inhibitor of Differentiation/DNA binding-1*) is a direct downstream target of BMP signaling, and the levels of *ID1* mRNA are widely used as a sensitive indicator for the status of BMP signaling. The function of ID1 in hepcidin expression and iron homeostasis is unknown. BMP6 is an essential cytokine for the induction of hepcidin expression *via* the BMP-signaling pathway in the liver (19, 20, 22, 23). Since these hepatoma cells do not detectably express most of the genes that are involved in iron homeostasis, these results suggest that in the absence of other iron regulatory proteins, inhibition of γ-secretase proteolysis of Neo1 is insufficient to impact hepcidin expression.

**Figure 5:**
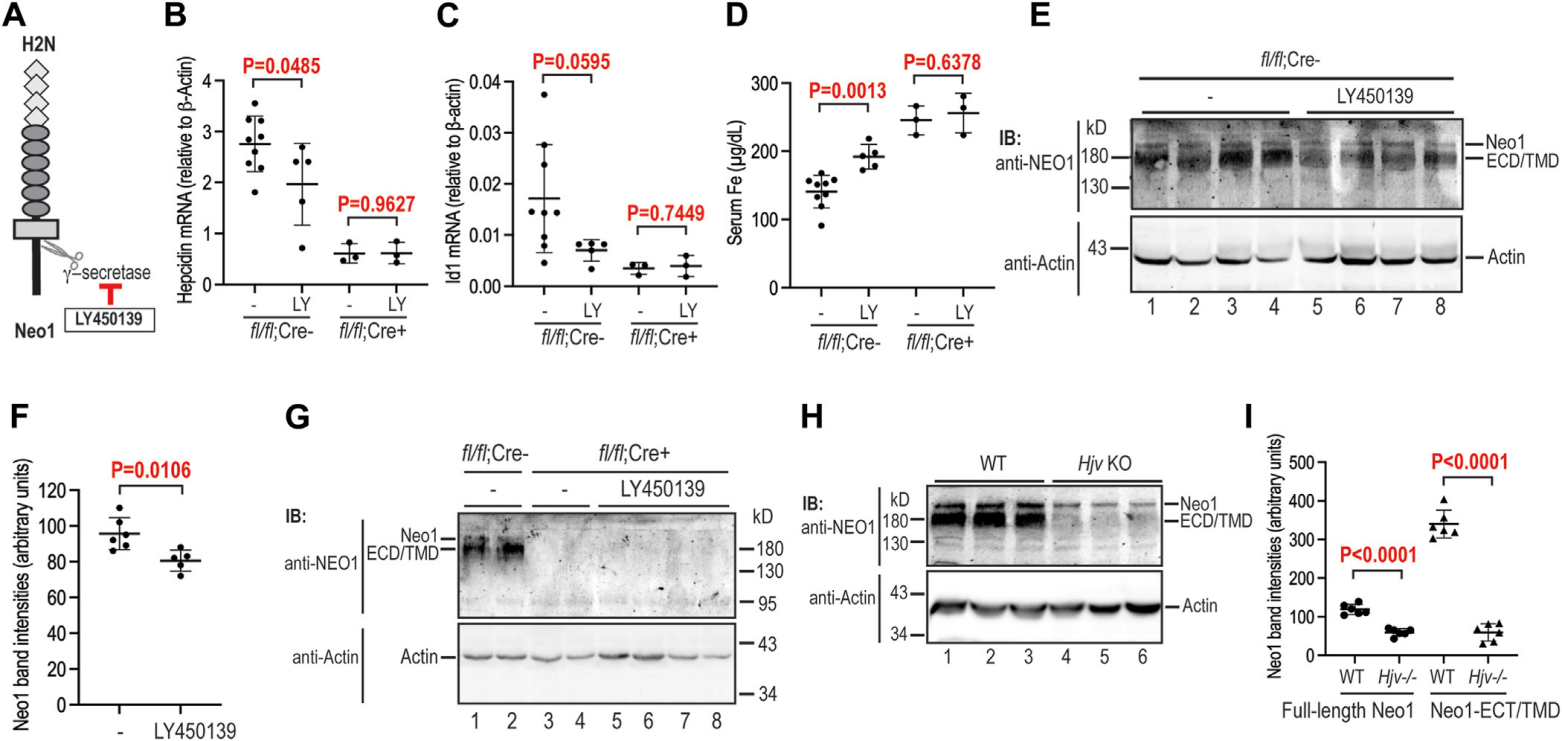
**Administration of γ-secretase inhibitor reduces hepatic hepcidin expression in mice.***A*, diagram of the target for γ-secretase inhibitor, LY450139. *B*-*D*, quantitative reverse transcription-PCR (qRT-PCR) analysis of hepatic *hepcidin* (*B*) and *Id1* (*C*) mRNA levels, as well as serum Fe concentrations (*D*). Eight-week-old *Neo1*^*fl/fl*^*;Alb-Cre*^*-*^ or *Neo1*^*fl/fl*^*;Alb-Cre*^+^ female mice were administered with LY450139 (LY; 30 mg/kg body weight) at 24 h (subcutaneously) and 6 h (intraperitoneally) before euthanasia. Injection with vehicle was used as controls (−). Each group consists of at least three animals. All qRT-PCR results are expressed as the amount relative to that of β-actin for each sample. *E*, representative images of Western blot analysis for endogenous Neo1 and β-actin in the liver membrane extracts (250 μg protein) from the WT *Neo1*^*fl/fl*^*;Alb-Cre*^*-*^ mice in B-D. *F*, quantification of Neo1-ECD/TMD bands in (*E*) (*top* panel; n ≥ 5). *G*, representative images of Western blot analysis for endogenous Neo1 and β-actin in the liver membrane extracts (250 μg protein) from *Neo1*^*fl/fl*^*;Alb-Cre*^−^ or *Neo1*^*fl/fl*^*;Alb-Cre*^+^ mice in *B*-*D*. *H*, representative images of Western blot analysis for endogenous Neo1 and β-actin in the liver membrane extracts from WT and *Hjv*^*−/−*^ mice. *I*, quantification of Neo1 and Neo1-ECD/TMD bands in (H) (*top panel*; n = 6). Data shown are means ± SD. Two-tailed student-T test was used to analyze the data for each genotype. The *p* values were presented.

The liver expresses γ-secretase (48, 49). We next tested whether inhibition of γ-secretase affects hepatic hepcidin expression in WT (*Neo1*^*fl/fl*^*;Alb-Cre*^*-*^) mice by systemic administration of LY450139 (Fig. 5*A*). LY450139 is a potent γ-secretase inhibitor with IC50 of ∼12 nM. It inhibits γ-secretase, but not α-secretase, proteolysis of Neo1 in Hep3B cells (Fig. 3, *B* and *C*). Because the Hjv-mediated hepcidin expression is associated with the increases in γ-secretase–cleaved Neo1-ECD/TMD in the liver (28), we reasoned that inhibition of γ-secretase activity would lead to a reduction of hepcidin expression. Since γ-secretase cleaves and regulates numerous membrane proteins (46), we examined the acute effects of LY450139 after two consecutive injections at 24-h (subcutaneously) and 6-h (intraperitoneally) before euthanasia in order to minimize the potential off-target effects. The administered dose for LY450139 (30 mg/kg) was chosen according to the previous *in vivo* studies (50). Injection of vehicle was used as controls. The hepatocyte-specific *Neo1* KO (*Neo1*^*fl/fl*^*;Alb-Cre*^*+*^) mice were included as additional controls. Indeed, administration of LY450139 resulted in a significant decrease in *hepcidin* mRNA and a trend decrease in *Id1* mRNA in WT *Neo1*^*fl/fl*^*;Alb-Cre*^*-*^ mice (Fig. 5, *B* and *C*). The trend decrease in *Id1* mRNA suggests that the decreased hepcidin expression resulted from reduced Bmp signaling. Consistently, a significant increase in serum iron concentrations was observed in these mice (Fig. 5*D*). As negative controls, no significant change was detected when LY450139 was administered to the *Neo1*^*fl/fl*^*;Alb-Cre*^+^ mice (Fig. 5, *B*–*D*). In agreement with our earlier studies (28), both full-length Neo1 and the truncated Neo1-ECD/TMD were detected in the liver membrane preparations of WT *Neo1*^*fl/fl*^*;Alb-Cre*^*-*^ mice by Western blot analysis with the latter as the predominant form (Fig. 5*E*; top panel, lanes 1–4). Administration of LY450139 resulted in decreases in the truncated Neo1-ECD/TMD band intensities in the liver membrane preparations of WT mice (Fig. 5, *E* and *F*). As negative controls, neither form of Neo1 was detectable in the liver membrane preparations of the hepatocyte-specific *Neo1* KO mice with or without administration of LY450139 (Fig. 5*G*; top panel; lanes 3–8). These results suggest that the decreased hepatic *hepcidin* mRNA levels by LY450139 in the WT *Neo1*^*fl/fl*^*;Alb-Cre*^*-*^ mice were likely attributed to the inhibition of γ-secretase proteolysis of Neo1 in the liver.

Additionally, we also compared the profiles of hepatic Neo1 levels between global *Hjv* KO (*Hjv*^*−/−*^) mice and the corresponding WT counterparts. Consistent with our earlier studies (28), Western blot analysis revealed ∼80% decrease of truncated Neo1-ECD/TMD and ∼50% reduction of full-length Neo1 in the liver membrane preparations of *Hjv*^*−/−*^ mice (Fig. 5, *H* and *I*). Since ablation of Hjv does not affect hepatic Neo1 mRNA levels (28), these results suggest that hepatic Hjv facilitates the generation of Neo1-ECD/TMD at least partially by preventing full-length Neo1 degradation. Since the reduction of Neo1-ECD/TMD alone by γ-secretase inhibitor, LY450139, was able to reduce hepcidin expression (Fig. 5, *B* and *E*), these observations suggest that the marked reduction of hepatic hepcidin expression in *Hjv*^*−/−*^ mice (28) most likely results from the lack of Neo1-ECD/TMD generation in the liver and imply that the γ-secretase proteolysis is an indispensable process for the function of hepatic Neo1 in hepcidin expression.

### Deletion of the Neo1 intracellular domain does not affect α-secretase proteolysis but abolishes its ability to induce hepcidin expression in mice

**Figure 6:**
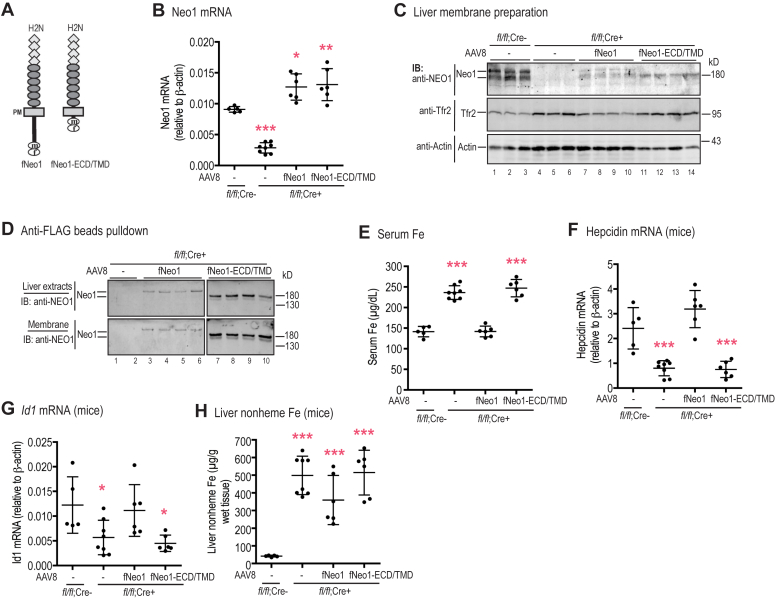
**Deletion of the intracellular domain abolishes Neo1 induction of hepcidin expression in *Neo1***^***fl/fl***^***;Alb-Cre***^**+**^ **mice.***A*, diagrams of mouse fNeo1 and fNeo1-ECD/TMD constructs. *B*, quantitative reverse transcription-PCR (qRT-PCR) analysis of hepatic *Neo1* mRNA from PBS-injected *Neo1*^*fl/fl*^*;Alb-Cre*^−^ male mice, PBS-injected *Neo1*^*fl/fl*^*;Alb-Cre*^+^ male mice (−), and *Neo1*^*fl/fl*^*;Alb-Cre*^+^ male mice transduced with AAV8-fNeo1 or fNeo1-ECD/TMD. *C*, representative images of Western blot analysis for fNeo1, fNeo1-ECD/TMD, Tfr2, and β-actin in the liver membrane preparation (250 μg protein). *D*, representative images of Western blot analysis for fNeo1 and fNeo1-ECD/TMD from the whole liver extracts (liver extracts) and the liver membrane preparation (membrane) of the *Neo1*^*fl/fl*^*;Alb-Cre*^+^ mice transduced with AAV8-fNeo1 or fNeo1-ECD/TMD. fNeo1 and fNeo1-ECD/TMD from ∼2 mg extract proteins were pulled down by using anti-FLAG affinity gel, followed by immunodetection (IB). *E*, serum iron (Fe) assay. *F*-*G*, qRT-PCR analysis of hepatic *hepcidin* and *Id1* mRNA. *H*, liver nonheme iron (Fe) assay. Each group consists of at least five animals. All qRT-PCR results are expressed as the amount relative to that of β-actin for each sample. Data shown are means ± SD. Since both *Neo1*^*fl/fl*^*;Alb-Cre*^*+*^ and the littermate *Neo1*^*fllwt*^*;Alb-Cre*^*-*^ control mice were generated by crossing *Neo1*^*fl/fl*^*;Alb-Cre*^*+/−*^male and *Neo1*^*fl/fl*^*;Alb-Cre*^*+/−*^ female on the C57BL/6J background and because expression of Alb-Cre alone does not affect iron homeostasis, one-way ANOVA and Tukey’s post-test were used to analyze the data relative to PBS-injected *Neo1*^*fl/fl*^*;Alb-Cre*^*-*^ mice. ∗, *p* < 0.05; ∗∗, *p* < 0.01; ∗∗∗, *p* < 0.001.

We next tested whether Neo1-ECD/TMD (Fig. 2, *A* and *B*) is the biologically functional form of Neo1 in the induction of hepcidin expression. The precise γ-secretase cleavage site in Neo1 is not known; therefore, we generated a fNeo1-ECD/TMD construct by deleting the coding sequence after Arg1170, the 10th amino acid after the transmembrane sequence, to ensure its proper integration into the membrane during biosynthesis. The coding sequence for the C-terminal FLAG/MYC epitope remained (Fig. 6*A*). In Hep3B cells, the transfected fNeo1-ECD/TMD behaved similarly to full-length fNeo1. Cell surface biotinylation studies indicate that it traffics to the plasma membrane (Fig. S5*A*). Both the α-secretase–cleaved forms, fNeo1-ECD and fNeo1-TMD (Fig. 2*A*), were readily detected in the conditioned medium (Fig. S5*A*) and in the cell extracts after anti-FLAG bead pulldown (Fig. S5*B*), respectively. These results indicate that deletion of Neo1 intracellular domain does not affect its ability to traffic to the plasma membrane and α-secretase proteolysis. When fNeo1-ECD/TMD or full-length fNeo1 was overexpressed in Hep3B cells, no significant increase in *hepcidin* or *ID1* mRNA was observed in either the presence, or the absence, of BMP6 ligands in the culture medium (Fig. S5, *C*–*E*). Similarly, increased expression of human NEO1 in HepG2 cells also failed to enhance *hepcidin* and *ID1* mRNA levels (Fig. S6). These results suggest that mice remain the best model to study the function of Neo1 in hepcidin expression.

We expressed fNeo1-ECD/TMD in the liver of hepatocyte-specific *Neo1* KO (*Neo1*^*fl/fl*^*;Alb-Cre*^*+*^) mice using AAV8 vectors in order to test whether the Neo1-ECD/TMD was sufficient to induce the Bmp-mediated signaling, thereby recovering hepcidin expression. Transduction of full-length fNeo1 was used as a positive control, and injection of PBS vehicle served as a negative control. Our previous studies demonstrated that administration of empty AAV8 vector had no evident impacts on hepatic *hepcidin* expression and iron homeostasis in mice (27, 51). This vector is able to specifically express the gene of interest in the hepatocytes, and the expression of the introduced gene is distributed in hepatocytes throughout the liver (52). Since AAV8-transduced complementary DNA (cDNA) rarely integrates into the genome, animals were euthanized for analysis 3 weeks after viral administration. The expression levels in the transduced livers were analyzed by qRT-PCR and Western blot. The *Neo1* mRNA levels in fNeo1 and fNeo1-ECD/TMD groups were modestly higher than those in PBS-injected WT *Neo1*^*fl/fl*^*;Alb-Cre*^−^ controls (Fig. 6*B*). The expressed proteins in liver extracts were confirmed by using anti-NEO1 and anti-FLAG antibodies (Fig. 6, *C* and *D*). Both fNeo1 and fNeo1-ECD/TMD migrated at the predicted molecular weights. Since both the *Neo1*^*fl/fl*^*;Alb-Cre*^+^ mice and the *Neo1*^*fl/fl*^*;Alb-Cre*^*-*^ controls were generated by crossing *Neo1*^*fl/fl*^*;Alb-Cre*^*+/−*^ male and *Neo1*^*fl/fl*^*;Alb-Cre*^*+/−*^ female on a C57BL/6J background and because expression of Alb-Cre alone displayed no evident effect on iron homeostasis (unpublished observations), the only differences between the animals used in the *in vivo* studies were the levels and forms of expressed Neo1.

Consistent with our previous studies (28), expression of exogenous fNeo1 was able to fully correct the high serum iron and low *hepcidin* mRNA status in *Neo1*^*fl/fl*^*;Alb-Cre*^+^ mice (Fig. 6, *E* and *F*). The increased *hepcidin* mRNA was induced by elevated Bmp-signaling as indicated by a significant increase in *Id1* mRNA (Fig. 6*G*). In agreement with the decrease in serum iron by fNeo1, the elevated Tfr2 returned to the comparable levels of WT *Neo1*^*fl/fl*^*;Alb-Cre*^*-*^ controls (Fig. 6*C*; panel 2, lanes 7–10). Tfr2 is a transmembrane protein that is expressed highly in hepatocytes (53, 54). It binds iron-bound transferrin (55, 56). Hepatic Tfr2 is stabilized by increased transferrin saturation (57, 58). Intriguingly, expression of similar levels of fNeo1-ECD/TMD in the liver of *Neo1*^*fl/fl*^*;Alb-Cre*^+^ mice displayed no significant effect on serum iron, hepatic *hepcidin* and *Id1* mRNA, Tfr2, or liver nonheme iron levels when compared with PBS-injected negative controls (Fig. 6, *B*–*H*). The liver is the major organ that stores the excess iron in the body. The levels of liver nonheme iron are widely used as an indicator of iron storage. These observations indicate that expression of fNeo1-ECD/TMD is unable to induce hepcidin expression in the liver and suggest that the intracellular domain of Neo1 is indispensable for its biological function. The unaffected α-secretase cleavage of the transfected fNeo1-ECD/TMD in Hep3B cells (Fig. S5*A*) implies that the α-secretase–cleaved Neo1-ECD is not directly involved in the induction of hepcidin expression.

### Studies of truncated Neo1 intracellular domain suggest that the function Neo1 requires a *de novo* γ-secretase proteolysis

**Figure 7:**
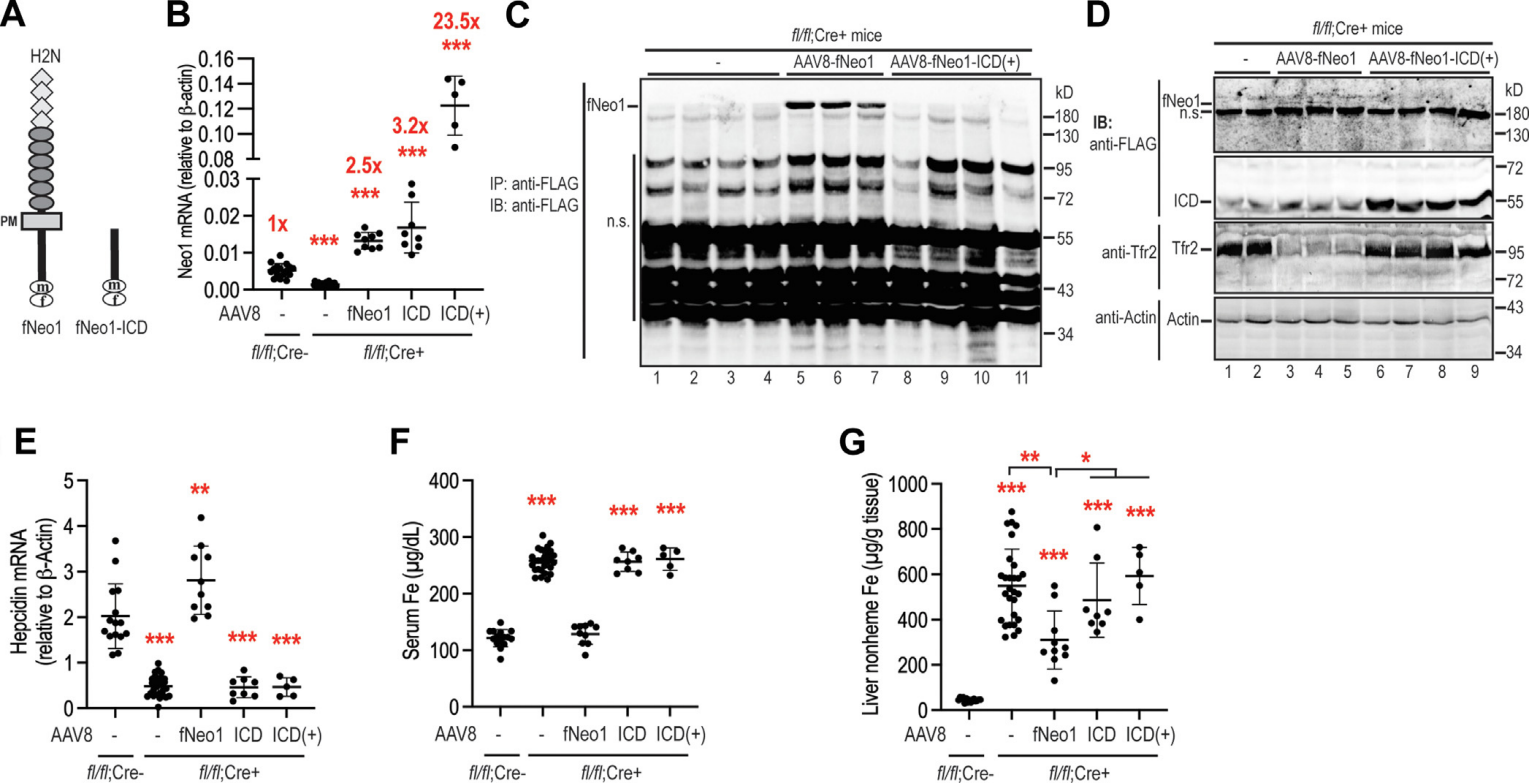
**Expression of Neo1-ICD is unable to correct the low hepcidin status in *Neo1***^***fl/fl***^***;Alb-Cre***^**+**^ **mice.***A*, diagrams of mouse fNeo1 and fNeo1-ICD constructs. *B*, quantitative reverse transcription-PCR (qRT-PCR) analysis of hepatic *Neo1* mRNA from PBS-injected *Neo1*^*fl/fl*^*;Alb-Cre*^−^ male mice, PBS-injected *Neo1*^*fl/fl*^*;Alb-Cre*^+^ male mice (−), and *Neo1*^*fl/fl*^*;Alb-Cre*^+^ male mice transduced with AAV8-fNeo1 at ∼3.5 × 10^10^ viral genome-particles per mouse or AAV8-fNeo1-ICD at ∼3.5 × 10^10^ or ∼2.8 × 10^11^ (+) viral genome-particles per mouse. *C*, representative images of Western blot analysis for fNeo1 and fNeo1-ICD from the whole liver extracts of *Neo1*^*fl/fl*^*;Alb-Cre*^+^ mice transduced with AAV8-fNeo1 or fNeo1-ICD (+) after anti-FLAG bead pulldown. *D*, representative images of Western blot analysis for FLAG, Tfr2, and β-actin in the whole liver extracts of *Neo1*^*fl/fl*^*;Alb-Cre*^+^ mice transduced with AAV8-fNeo1 or fNeo1-ICD (+). *E*, qRT-PCR analysis of hepatic *hepcidin* mRNA. *F*, serum Fe assays. *G*, liver nonheme Fe assay. Each group consists of at least eight animals. All qRT-PCR results are expressed as the amount relative to that of β-actin for each sample. Data shown are means ± SD. One-way ANOVA and Tukey’s post-test were used to analyze the data relative to PBS-injected *Neo1*^*fl/fl*^*;Alb-Cre*^*-*^ mice. ∗, *p* < 0.05; ∗∗, *p* < 0.01; ∗∗∗, *p* < 0.001.

To test whether the function of Neo1 is mediated through the γ-secretase–cleaved Neo1-ICD as suggested by the studies in other models (38, 39), we analyzed its biological function in hepatic hepcidin expression by transduction of fNeo-ICD into the liver of hepatocyte-specific *Neo1* KO *Neo1*^*fl/fl*^*;Alb-Cre*^+^ mice. The fNeo1-ICD construct was generated by deleting the sequence encoding Neo1 amino acids 2 to 1157 (Fig. 7*A*) on the basis of earlier studies (38). Transfected fNeo1-ICD in Hep3B cells migrated at the predicted molecular weight (∼55 kD) similarly to γ-secretase–cleaved ICD in SDS-PAGE (Fig. 3*B*; panel 1; lanes 10/13/14). The stability of transfected fNeo1-ICD was comparable to those of full-length fNeo1 in Hep3B cells by using cycloheximide (100 μg/ml) to block protein biosynthesis (Fig. S7).

We expressed fNeo-ICD in the liver of *Neo1*^*fl/fl*^*;Alb-Cre*^+^ mice by intraperitoneal administration of AAV8-fNeo1-ICD vectors at ∼3.5 × 10^10^ or ∼2.8 × 10^11^ viral genome-particles per mouse. Mice with administration of AAV8-fNeo1 vectors at ∼3.5 × 10^10^ viral genome-particles per mouse were included as positive controls. As shown in Fig. 7*B*, qRT-PCR analysis revealed that the mRNA levels of exogenously administered fNeo1-ICD were about 3.2 and 23.5-fold higher in the low and high dosage groups, respectively, than those of endogenously expressed Neo1 mRNA in the WT *Neo1*^*fl/fl*^*;Alb-Cre*^*-*^ controls. The protein levels of transduced fNeo1-ICD in the liver were first examined by Western blot analysis after pull-down with anti-FLAG beads. While the transduced full-length fNeo1 was readily detectable by using an anti-FLAG antibody (Fig. 7*C*; lanes 5–7), no distinct fNeo1-ICD band was observed because of the strong nonspecific overlapping signals (Fig. 7*C*; lanes 8–11). Therefore, the whole liver extracts were used directly for analysis. In comparison with the PBS-injected *Neo1*^*fl/fl*^*;Alb-Cre*^+^ controls, a strong band migrating at ∼55 kD was observed in all animals of the high AAV8-fNeo1-ICD dosage group [AAV8-fNeo1-ICD(+)] (Fig. 7*D*; panel 2; lanes 1/2 *versus* 6–9). The fNeo1-ICD protein in the low AAV8-fNeo1-ICD dosage group fell below the limit of immunodetection (data not shown). Together, these results suggest that the transduced fNeo1-ICD was indeed expressed in the liver.

Functional studies show that in contrast to the full-length fNeo1, expression of neither a lower nor a high level of fNeo1-ICD was able to correct the low hepatic hepcidin expression and high serum iron concentrations and to reduce liver nonheme iron levels in hepatocyte-specific *Neo1* KO *Neo1*^*fl/fl*^*;Alb-Cre*^+^ mice (Fig. 7, *E*–*G*). In agreement with the unchanged serum iron concentrations by fNeo1-ICD (Fig. 7*F*), hepatic Tfr2 levels remained high when compared with the PBS control group (Fig. 7*D*; lanes 1/2 *versus* 6–9). As expected, the reduced serum iron by fNeo1 resulted in a significant decrease of hepatic Tfr2 (Fig. 7*D*; lanes 1/2 *versus* 3–5). These results suggest that expression of this form of Neo1-ICD is unable to induce hepcidin expression. In conjunction with the above *in vivo* studies of γ-secretase inhibitor and fNeo1-ECD/TMD (Figs. 5 and 6), these observations imply that the function Neo1 might need a *de novo* γ-secretase proteolysis.

### Deletion of the Neo1 Ig-like domains compromises its ability to induce hepcidin expression

**Figure 8:**
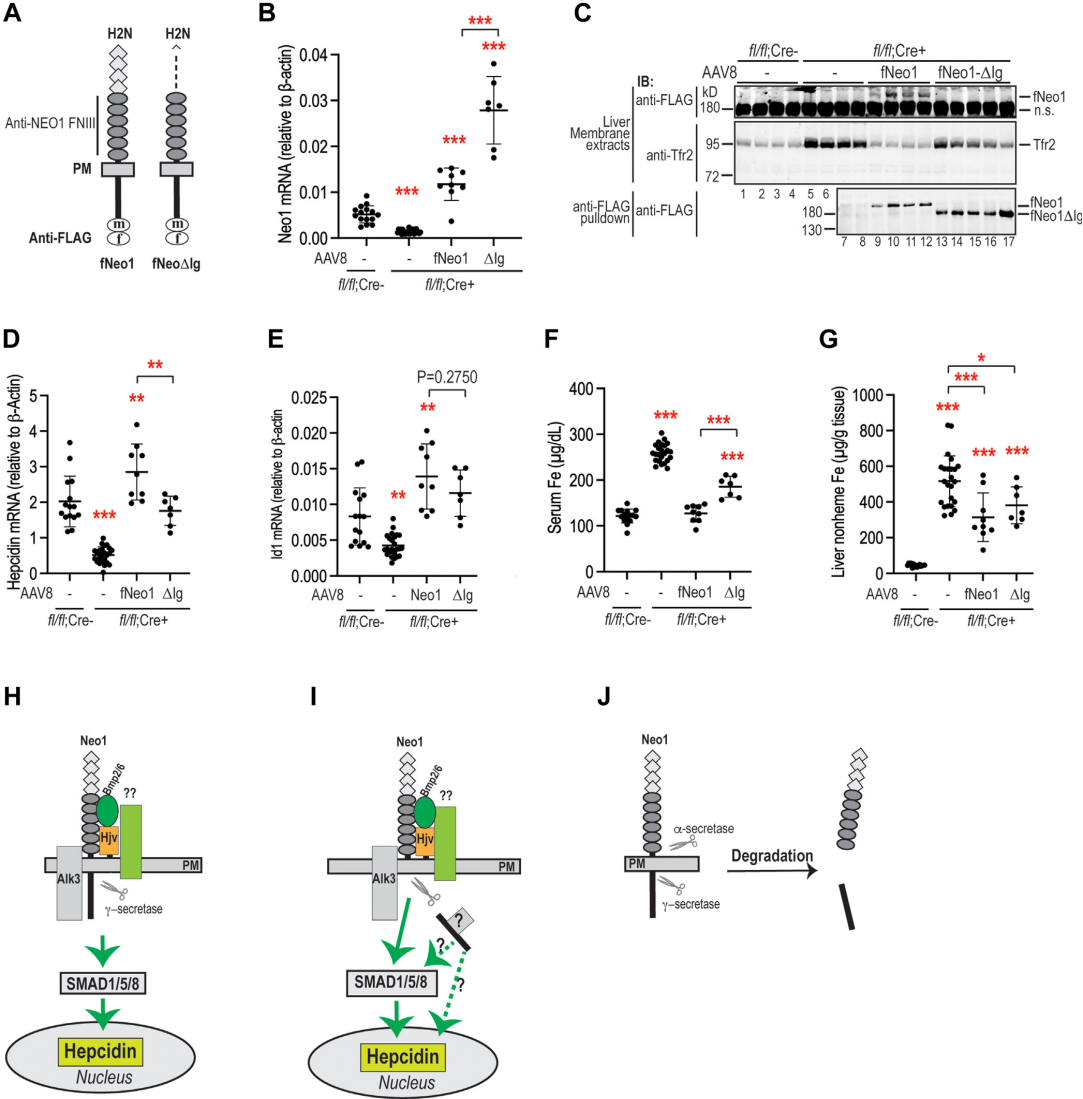
**Deletion of the Neo1 Ig-like domains compromises its ability to induce hepcidin expression.***A*, diagrams of mouse fNeo1 and fNeo1^ΔIg^ constructs. *B*, qRT-PCR analysis of hepatic *Neo1* mRNA from PBS-injected *Neo1*^*fl/fl*^*;Alb-Cre*^−^ male mice, PBS-injected *Neo1*^*fl/fl*^*;Alb-Cre*^+^ male mice (−), and *Neo1*^*fl/fl*^*;Alb-Cre*^+^ male mice transduced with AAV8-fNeo1 and male fNeo1^ΔIg^. *C*, representative images of Western blot analysis for transduced fNeo1, fNeo1^ΔIg^, and endogenous Tfr2 from the mice as described above in (*B*). Images in panels-1/2 were derived from the immunodetection of liver membrane extracts (250 μg protein). The image in panel-3 was obtained from the same membrane extracts, except that ∼2 mg extract proteins from each sample were first subjected to pull down by using anti-FLAG affinity gel, followed by immunodetection with an anti-FLAG antibody. *D*-*E*, qRT-PCR analysis of hepatic *hepcidin* and *Id1* mRNA. *F*, serum iron (Fe) assay. *G*, liver nonheme iron (Fe) assay. Each group consists of at least seven animals. All qRT-PCR results are expressed as the amount relative to that of β-actin for each sample. Data shown are means ± SD. One-way ANOVA and Tukey’s post-test were used to analyze the data relative to PBS-injected *Neo1*^*fl/fl*^*;Alb-Cre*^*-*^ mice. In addition, the analyzed results between fNeo1 and fNeo1^ΔIg^ groups were also presented. ∗, *p* < 0.05; ∗∗*p* < 0.01; ∗∗∗, *p* < 0.001. *H*-*J*, models for the induction of hepcidin expression by hepatocyte Neo1 *via* its interaction with Hjv, Alk3, and other hepcidin-inducing proteins. PM, plasma membrane; n.s., nonspecific band.

Our earlier co-immunoprecipitation studies indicate that NEO1 interacts with ALK3 and that this interaction requires the N-terminal Ig-like domains of NEO1 (27). Since Alk3 is an essential type-I BMP receptor for hepatic hepcidin expression (16), we next examined whether these Ig-like domains are involved in Neo1 induction of hepcidin expression. We generated an fNeo1^ΔIg^ construct by deletion of the coding sequence for Ser40-Leu469 with no disruption of the N-terminal signal sequence (Fig. 8*A*). In transfected Hep3B cells, deletion of Ig-like domains did not affect its trafficking to the plasma membrane (Fig. S8*A*). Anti-FLAG pulldown analysis detected comparable levels of fNeo1-TMD/ICD (α-secretase–cleaved products) and fNeo1-ICD (γ-secretase–cleaved products) between full-length fNeo1 and fNeo1^ΔIg^ (Fig. S8*B*). No α-secretase–cleaved fNeo1^ΔIg^-ECD was detected in the conditioned medium (Fig. S8*B*), which likely resulted from its rapid degradation. Thus, these results suggest that lack of the Ig-like domains does not affect the Neo1 cleavage by either α- or γ-secretase.

We employed similar approaches as described above to test the biological function of Neo1^ΔIg^ by transduction into the liver of *Neo1*^*fl/fl*^*;Alb-Cre*^+^ mice. The expressed fNeo1^ΔIg^ mRNA levels were about 2.5-fold higher than the full-length fNeo1 group and about 5-fold higher than the endogenous Neo1 mRNA in the WT *Neo1*^*fl/fl*^*;Alb-Cre*^*−/−*^ mice (Fig. 8*B*). Since a nonspecific binding protein in the liver membrane extracts was overlapping with fNeo1^ΔIg^ in the Western blot probed with anti-FLAG antibody (Fig. 8*C*; panel 1), the fNeo1^ΔIg^ protein was detected after the anti-FLAG bead pulldown. The relative protein levels of fNeo1 and fNeo1^ΔIg^ were proportional to their mRNA levels (Fig. 8, *B* and *C*, panel-3, lanes 9–12 *versus* 13–17). Interestingly, fNeo1^ΔIg^ displayed a compromised ability to induce hepcidin expression. Expression of higher levels of fNeo1^ΔIg^ was unable to enhance hepcidin expression as robustly as fNeo1 (Fig. 8*D*). This is likely due to the reduced induction of Bmp-signaling as indicated by the lower Id1 mRNA levels (Fig. 8*E*). In line with the hepatic hepcidin mRNA levels, serum iron concentrations in fNeo1 group were decreased to that of WT *Neo1*^*fl/fl*^*;Alb-Cre*^*-*^ group, whereas the fNeo1^ΔIg^ group only displayed a moderate reduction (Fig. 8*F*). In agreement with serum iron, hepatic Tfr2 levels in Neo1^ΔIg^ group fell between the fNeo1 and PBS-injected *Neo1*^*fl/fl*^*;Alb-Cre +* groups (Fig. 8*C*; panel 2). The liver nonheme iron levels showed no significant difference between fNeo1 and fNeo1^ΔIg^ groups (Fig. 8*G*). Together, these results indicate that fNeo1^ΔIg^ displays a compromised ability to induce hepcidin expression and suggest that the Ig-like domains are also necessary for the function of Neo1.

## Discussion

This study explored the potential mechanisms by which hepatic Neo1 induces hepcidin expression. *In vitro* studies indicate that the Hjv-increased Neo1-ECD/TMD in the liver (28) was derived from a direct γ-secretase proteolysis of full-length Neo1 and that the generation of Neo1-ECD/TMD was increased by inhibition of α-secretase cleavage of Neo1 ectodomain and by Neo1 association with Alk3. *In vivo* studies suggest that the Neo1 induction of hepcidin requires a *de novo* γ-secretase proteolysis. Additional studies revealed that in addition to the Hjv-binding domains, the function of Neo1 also needed its C-terminal intracellular domain and its N-terminal Ig-like domains. Together, our data support the idea that hepatic Neo1 induction of hepcidin is initiated as a full-length form and requires a *de novo* γ-secretase cleavage of Neo1’s cytoplasmic domain for its optimal stimulation of hepcidin expression.

NEO1 can be cleaved by α- and γ-secretases (36, 37, 38, 39). Both proteases are expressed in the liver (48, 49, 59). We used hepatoma cells and specific inhibitors to determine whether the Hjv-increased Neo1-ECD/TMD in the liver (28) is generated by preventing the proteosome-mediated degradation of cleaved Neo1. Our data validated that Neo1 underwent cleavage by the endogenous α- and γ-secretases independently of Hjv. We obtained evidence supporting that the membrane-associated Neo1-ECD/TMD was generated by a direct γ-secretase proteolysis of full-length Neo1. In hepatoma cells, we found that γ-secretase is the predominant protease to release the Neo1-ICD. Although majority of the transfected Neo1 underwent a sequential cleavage process similarly to other γ-secretase substrates (46, 47), yet our PSEN1/2 knockdown and inhibitor studies strongly indicate that a fraction of full-length Neo1 bypasses this classical pathway and can be cleaved directly by γ-secretase without a prior α-secretase cleavage. This latter process leads to the generation of truncated Neo1-ECD/TMD. In agreement with this observation, a similar form of truncated Neo1 by γ-secretase cleavage is reported in the neuron of mice (40). Importantly, a recent study also shows that the full-length amyloid precursor-like protein 1 (APLP1) is cleaved by γ-secretase without previous ectodomain shedding (60). However, different from the predominant membrane association of Neo1-ECD/TMD, the γ-secretase–cleaved APLP1 is mainly detected as a released soluble form in the conditioned medium of transfected cells (60). We speculate that this discrepancy between Neo1 and APLP1 is likely due to the difference of their transmembrane domain sequences. In contrast to the Neo1-ECD/TMD as a predominant form of Neo1 in the liver of WT mice (Fig. 5*H*) (28), the relatively low levels of Neo1-ECD/TMD detected in hepatoma cells are likely attributed to the limitation of overexpression model used in the study, as well as the lack of Hjv and other hepcidin-inducing proteins.

We found that the Hjv-mediated increases of Neo1-ECD/TMD in the liver (28) could be partially recapitulated by inhibition of α-secretase proteolysis. In the absence of Hjv, inhibition of α-secretase activity was able to increase the levels of a truncated Neo1-ECD/TMD on the cell surface. This form of Neo1-ECD/TMD is analogous to the Hjv-stabilized Neo1-ECD/TMD in the liver (28), because both are membrane-associated, lack an intracellular domain, and migrate at a similar molecular weight in SDS-PAGE. Earlier studies showed that the α-secretase cleavage of Neo1 ectodomain abolishes the RGMa-induced Neo1 signaling in neurons (36). Since the Hjv-mediated hepcidin expression is associated with the increases of Neo1-ECD/TMD levels in the liver (28), these observations imply that the Hjv-mediated hepcidin expression is at least partially achieved by blocking α-secretase proteolysis of Neo1 ectodomain.

Neo1 binds Hjv, Alk3, and netrin-1 (3, 4, 27, 32). Our studies in hepatoma cells demonstrated that the generation of Neo1-ECD/TMD is facilitated by the membrane-associated Neo1-binding partner, Alk3, and that the glycosylphosphatidylinositol-anchored Hjv protects Neo1 from netrin-1–mediated degradation of Neo1. The α-secretase cleavage site in Neo1 is predicted to fall within the Neo1 domains that bind Hjv and Netrin-1. In contrast to our initial predictions from the studies with α-secretase inhibitors and the earlier reports showing that RGMa, a close family member of Hjv, promotes γ-secretase–mediated proteolysis of NEO1 in cell lines and zebrafish (38, 39), our data indicate that the Hjv binding to the α-secretase cleavage region in Neo1 alone was insufficient to inhibit α-secretase cleavage of Neo1 and to increase the generation of Neo1-ECD/TMD. We speculate that this is likely due to the facts that the hepatoma cells do not express many of the genes involved in iron homeostasis (28). In line with the Hjv studies, the association with netrin-1 alone was also unable to block α-secretase proteolysis of Neo1. Rather, the netrin-1 binding resulted in a rapid proteasomal degradation of Neo1. These observations suggest that Neo1 association with a single binding partner is incapable of blocking α-secretase proteolysis.

Both HJV and netrin-1 can simultaneously bind to NEO1 (32). Further studies revealed that Hjv was able to largely prevent netrin-1–mediated Neo1 degradation. Netrin-1 is expressed primarily by the neurons, and it is detectable (∼673.5 pg/ml) in the circulation of healthy adults (61). Interestingly, a recent study shows that hepatic inflammation markedly upregulates the production of netrin-1 in the liver (33). These observations imply that the Neo1 association with a membrane-tethered partner, Hjv, can protect Neo1 from degradation, which are elicited by the netrin-1 in the circulation and by the inflammation-induced netrin-1 in the liver. This idea is strongly supported by the studies with Alk3. We found that co-expression of Alk3 with Neo1 was able to markedly increase full-length Neo1 and fNeo1-ECD/TMD on the cell surface. Intriguingly, Alk3 also enhanced α- and γ-secretase proteolysis of Neo1 in Hep3B cells. These data suggest that the Alk3-increased generation of Neo1-ECD/TMD is likely achieved by stabilizing the full-length Neo1. This is consistent with the *in vivo* studies showing higher levels of both full-length Neo1 and truncated Neo1-ECD/TMD in the liver of WT mice when compared with *Hjv*^−/−^ mice (Fig. 5*H*) (28). Hepatocyte HJV acts as a co-receptor for BMP6 and uses two type-I BMP receptors, ALK2 and ALK3, to robustly stimulate hepcidin expression *via* the BMP-signaling pathway (14, 16, 22, 23, 25). Previous studies show that Hjv can bind multiple key components in the hepcidin-inducing pathway, including ALK2, ALK3, BMP6, the hemochromatosis protein, TfR2, as well as Neo1 (3, 4, 28, 62, 63) and indicate that Neo1 induces hepcidin expression by acting as a scaffold to facilitate the formation of a key hepcidin-inducing complex (3, 4, 16, 27). Thus, the data gained from this and earlier studies (3, 4, 16, 27, 38, 39) favor the ideas that the Hjv-mediated generation of Neo1-ECD/TMD in the liver (28) results from the formation of this complex and that this complex facilitates the direct γ-secretase proteolysis by preventing Neo1 degradation and by blocking α-secretase–mediated Neo1 ectodomain shedding.

We examined the effects of γ-secretase inhibition on hepcidin expression in mice. Results show that administration of the potent specific γ-secretase inhibitor, LY450139, was indeed able to significantly decrease hepcidin expression in WT mice, but not in the negative control *Neo1*^*fl/fl*^*;Alb-Cre*^+^ mice that lack hepatocyte Neo1. These data ruled out the potential off-target effects. Importantly, this decrease in hepcidin expression in WT mice was associated with a concomitant reduction in hepatic Neo1-ECD/TMD. These results are consistent with our earlier findings, which showed a positive correlation of the hepcidin expression with the levels of γ-secretase–cleaved Neo1-ECD/TMD in the liver (28). In agreement with this idea, a recent study reported that the γ-secretase cleavage of Neo1 is involved with the seizure-induced hippocampal necroptosis in mice. And prevention of Neo1 truncation by γ-secretase inhibitor can protect against NMDA-induced excitotoxicity in cultured neurons (40). Together, these observations support the idea that the γ-secretase proteolysis is an essential process for the function of hepatic Neo1 in iron homeostasis.

The γ-secretase proteolysis of Neo1 generates Neo1-ECD/TMD and Neo1-ICD (Fig. 2). On the basis of the positive association of hepatic hepcidin expression with the levels of γ-secretase–cleaved Neo1-ECD/TMD in the liver (28), we initially hypothesized that the Neo1-ECD/TMD is the biologically functional form. We generated the predicted Neo1-ECD/TMD construct by deleting most of its coding sequence for the Neo1 intracellular domain. Our data validated that the transfected fNeo1-ECD/TMD trafficked onto the plasma membrane and underwent α-secretase cleavage of its ectodomain similar to full-length fNeo1. This result indicates that the expressed fNeo1-ECD/TMD was properly folded. Surprisingly, expression of fNeo1-ECD/TMD in *Neo1*^*fl/fl*^*;Alb-Cre*^+^ mice was unable to correct the low hepcidin expression and high serum iron status, which is in contrast to a full correction by expressing comparable levels of full-length fNeo1. These observations have important implications. On one hand, they suggest that only the full-length Neo1 is the biologically functional form and that the γ-secretase–cleaved Neo1-ECD/TMD is a biologically inactive form. On the other hand, these findings also imply that the function of Neo1 could be mediated *via* the γ-secretase–cleaved Neo1-ICD. Earlier studies show that NEO1-ICD can translocate into the nucleus and regulate gene transcription in cell lines and zebrafish (38, 39). Nevertheless, these data strongly indicate that the intracellular domain of Neo1 is essential for its biological function.

The Neo1-ICD consists of ∼337 amino acids (64) and it contains a predicted nuclear localization motif (38, 39). We generated an fNeo1-ICD construct on the basis of earlier studies (38). In contrast to our prediction, expression of fNeo1-ICD was also unable to correct the low hepcidin expression and high serum iron status in *Neo1*^*fl/fl*^*;Alb-Cre*^+^ mice, which is similar to our *in vivo* studies for fNeo1-ECD/TMD. Although we cannot rule out the possibility that the expressed fNeo1-ICD or fNeo1-ECD/TMD is different from the endogenously cleaved forms, these findings imply that the function of Neo1 could be initiated as a full-length form and that both its extracellular and intracellular domains are required to form a hepcidin-inducing complex with other components. In this scenario, only the *de novo* γ-secretase–cleaved fNeo1-ECD/TMD and fNeo1-ICD are able to induce hepcidin expression. This idea was supported by our *in vivo* studies using the specific γ-secretase inhibitor.

Additional studies showed that the N-terminal Ig-like domains of Neo1 are also important for its biological function. Deletion of these domains compromised the ability of Neo1 to induce hepcidin mRNA in mice. Our earlier studies suggest that these Ig-like domains are involved in the interaction with ALK3 (27). Together with the findings that Hjv binds to the FNIII 5 to 6 domains of Neo1 (1, 2, 3, 4), these observations imply that a fully function Neo1 requires the interactions with both Hjv and Alk3. As a result, we speculate that the entire Neo1 ectodomain is involved in the assembly of Hjv-containing complex to enhance hepcidin expression in hepatocytes.

Taken together, our data support the idea that the function of hepatocyte Neo1 requires both its extracellular and intracellular domains as well as a *de novo* γ-secretase proteolysis in an Hjv-dependent manner. Based on our current data and previous reports (26, 27, 28, 38, 39, 40, 63, 65), we propose models for Neo1 induction of hepcidin expression. First, full-length Neo1 acts as a scaffold to facilitate the formation of a hepcidin-inducing complex, which induces hepcidin expression through the SMAD1/5/8-mediated BMP-signaling pathway (Fig. 8*H*). Alternatively, upon formation of this hepcidin-inducing complex, it will trigger γ-secretase proteolysis of Neo1-ICD. In this scenario, the released Neo1-ICD will translocate to the nucleus to induce hepcidin expression by facilitating the BMP signaling and/or through a Neo1-specific pathway, and the Neo1-Hjv–containing complex on the plasma membrane will induce hepcidin expression through the Hjv-mediated BMP-signaling pathway (Fig. 8*I*). Lack of the Hjv binding will lead to a rapid degradation of Neo1 (Fig. 8*J*). Future studies will test these models.

## Experimental procedures

### cDNA constructs

Mouse Neo1 ORF (NM_008684) with a C-terminal FLAG/MYC epitope (fNeo1) in pCMV6 vector (#MR226235) and mouse Alk3 ORF (NM_009758.3) with a C-terminal FLAG/MYC epitope (fAlk3) in pCMV6 vector (#MR227586) were obtained from OriGene Technologies Inc. We obtained pEGFP-N1 plasmid from Clontech. We generated the pCMV6-fNeo1-ECD/TMD, fNeo1-ICD, and fNeo1^ΔIg^ constructs by using pCMV6-fNeo1 as a template, the primers listed in Table S1, and the QuikChange site-directed mutagenesis kit (Agilent). The mouse Hjv construct with an N-terminal 3xFLAG epitope in pCMV9 vector (pCMV9-fHjv) was generated in previous studies (27). All sequences were verified by DNA sequencing.

### Cell lines, transfection, and biotinylation of cell surface proteins

Hep3B cells were obtained from ATCC. We used Hep3B cells and transient transfection of pCMV6-fNeo1, fNeo1-ECD/TMD, fNeo1-ICD, fNeo1^ΔIg^, or empty vector with Lipofectamine-3000 (Invitrogen) to test the cell surface localization of fNeo1, fNeo1-ECD/TMD, and fNeo1^ΔIg^ by biotinylation; the α/γ-secretase cleavage of fNeo1, fNeo1^ΔICD^, and fNeo1^ΔIg^; as well as the effects of fNeo1, fNeo1-ECD/TMD, and γ-secretase inhibitors on hepcidin expression. To study the effects of Hjv and Alk3 on α/γ-secretase cleavage of fNeo1, Hep3B cells were cotransfected with pCMV6-fNeo1/pEGFP-N1 (EGFP), pCMV9-fHjv/EGFP, pCMV6-fNeo1/pCMV9-fHjv, pCMV9-fAlk3/EGFP, or pCMV6-fNeo1/pCMV9-fAlk3 construct DNA at 1:1 ratio. Cotransfection with EGFP was to assure that all transfected cells expressed similar amounts of exogenous protein. The HepG2 cells that were stably transfected with full-length human NEO1 (HepG2-NEO1) or empty vector (HepG2-Ctrl) were generated in our previous studies (37). We used Hep3B and HepG2 cells to study the effects of γ-secretase inhibitors on hepcidin expression. The inhibitors used in the studies include α-secretase inhibitors, TAPI-1 (#B4686; APE × BIO) and Aderbasib (INCB007839; #HY-10293; MedChemExpress); γ-secretase inhibitors, DAPT (D5942; Sigma-Aldrich) and LY450139 (SML1938; Sigma-Aldrich); and proteasome inhibitor, MG132 (#47490; EMO Millipore Corp). BMP6 (#507-BP) and recombinant mouse netrin-1 with 10-His tag (#1109-N1) were purchased from R&D Systems.

The biotinylation of cell surface proteins was performed on ice. Briefly, transfected Hep3B cells in a 6-well plate were washed three times with ice-cold PBS + buffer (PBS with 0.5 mm CaCl_2_ and 1 mm MgCl_2_) and incubated with 0.25 mg/ml EZ-Link Sulfo-NHS-SS-Biotin (Thermo Fisher Scientific) in PBS + buffer for 30 min. The reaction was quenched by washing the cells five-times with ice-cold PBS buffer containing 0.1% glycine (pH 7.6). Cells were solubilized, and cell lysate was cleared by centrifugation at 16,000*g* for 5 min. Biotinylated proteins were isolated with streptavidin resin (G Biosciences). Bound proteins were eluted with 1x Laemmli buffer and subjected to SDS-PAGE and immunodetection by using anti-NEO1, FLAG, Na^+^K^+^ ATPase, and β-actin antibodies.

To detect the α-secretase–cleaved fNeo1 ectodomain in the conditioned medium (CM), the complete medium (MEM/10% FBS) was changed to Opti-MEM/1% FBS and incubated for the indicated time periods in the figure legends before collection for analysis. Proteins in ∼600 μl of CM were precipitated by using 6% TCA, followed by resuspending the protein pellets with 1x Laemmli buffer and subjecting to SDS-PAGE and immunodetection. To detect the γ-secretase–cleaved fNeo1-ICD, Hep3B cells were transiently transfected with pCMV6-fNeo1 and fNeo1-ICD constructs. At about 40 h after transfection, cells were incubated with 10 μM MG-132 for about 7 h to inhibit the turnover of the cleaved fNeo1-ICD. About 90% of cell lysate was subjected to pull-down using anti-FLAG affinity gel (Sigma #A2220), followed by elution using the 3xFLAG peptide at ∼200 μg/ml (Sigma F4799). The eluted proteins and ∼10% of input lysate were then subjected to SDS-PAGE and immunodetection by using specific antibodies.

### Knockdown of endogenous PSEN1 and 2

The SMARTpool siRNAs specific for human PSEN1 and 2 (Dharmacon) were used to simultaneously knockdown both PSEN1 and 2 in Hep3B cells as previously described for NEO1 knockdown (66). RNAiMAX reagent (Invitrogen) was used for the transfection. The negative control siRNA was purchased from Santa Cruz Biotechnology, Inc (#sc-37007). siRNA transfection was conducted in 6-well plates in complete medium. At about 16-h after siRNA transfection, cells were transfected with either pCMV6-fNeo1 or pEGFP-N1 (EGFP) control. Analysis was performed at ∼70 h after siRNA transfection.

### Subcellular protein fractionation

We used the Subcellular Protein Fractionation Kit for Cultured Cells from Thermo Fisher Scientific (#78840) to isolate the membrane proteins and cytoplasmic proteins from fNeo1-expressing Hep3B cells (∼5 × 10^6^) after incubation with 50 μM Aderbasib for ∼16 h. All isolated proteins were subjected to SDS-PAGE and Western blot analysis.

### Animal studies

All animal procedures were approved by OHSU Institutional Animal Care and Use Committee (IACUC). Homozygous hepatocyte-specific conditional *Neo1* KO (*Neo1*^*fl/fl*^*;Alb-Cre*^*+*^) mice and littermate *Cre*^*–*^ controls were generated by crossing *Neo1*^*fl/fl*^*;Alb-Cre*^*+/−*^ male and *Neo1*^*fl/fl*^*;Alb-Cre*^*+/−*^ female on a C57BL/6J background (28). Eight-week-old *Neo1*^*fl/fl*^*;Alb-Cre*^+^ male mice were intraperitoneally injected with AAV8-fNeo1 and fNeo1-ECD/TMD at ∼3.5 × 10^10^ viral genome-particles per mouse, fNeo1-ICD at ∼3.5 × 10^10^ or ∼2.8 × 10^11^ viral genome-particles per mouse, or AAV8-fNeo1^ΔIg^ at ∼7 × 10^10^ viral genome-particles per mouse. Injection of PBS vehicle was included as a control. Our previous studies showed that administration of AAV8 empty vector had no evident effect on hepatic hepcidin expression and iron homeostasis in mice (27, 51). Mice were euthanized for analysis at 3 weeks after injection for analysis. Age, gender, and background-matched *Cre*^*-*^ littermates were included as additional controls. To study the effects of γ-secretase inhibitor, eight-week-old *Neo1*^*fl/fl*^*;Alb-Cre*^−^ or *Neo1*^*fl/fl*^*;Alb-Cre*^+^ female mice were injected with LY450139 (30 mg/kg body weight) at 24 h (subcutaneously) and 6 h (intraperitoneally) before euthanasia. Injection with vehicle was used as controls. All mice were fed a PicoLab Laboratory Rodent Diet-5L0D containing 240-ppm iron (LabDiet).

We obtained the *Hjv*^*−/−*^ mice on 129/SvEvTac background from Dr Nancy Andrews (Duke University). The liver tissues were collected from eight-week-old male *Hjv*^*−/−*^ mice and WT 129/SvEvTac male mice for Western blot analysis of hepatic Neo1.

### Serum iron assay

Serum iron concentration was detected by using a Pointe Iron/TIBC Reagent Set (Pointe Scientific).

### Tissue nonheme iron assays

Tissue nonheme iron levels were determined as previously described (10) with the following modifications. Briefly, 50 to 150 mg wet tissues were digested in 250 to 750 μl of acid buffer at 65 °C for 72 h. The supernatant was collected by centrifugation at 10,000*g* for 5 min, followed by the addition of chromogen (1.86 mM bathophenanthroline sulfonate, 143 mM thioglycolic acid in water) and OD measurement at 535 nm. Each sample was measured twice in triplicate. Iron concentration is expressed as micrograms of iron per gram of wet tissue.

### qRT-PCR

Total RNA from mouse liver tissues, Hep3B cells, and HepG2 cells was extracted using a NucleoSpin RNA kit (Macherey-Nagel). cDNA was synthesized using Oligo deoxythymidine primers (Invitrogen) and M-MLV reverse transcriptase (Invitrogen). qRT-PCR analysis was carried out in triplicate on each sample using the Power SYBR Green PCR master mix in a QuantStudio 12K Flex qPCR System (Thermo Fisher Scientific). All primer sets used in these studies (Table S2) were validated against the reference primers (β-actin) to ensure approximately equal efficiencies of amplifications as previously described (67). The abundance of *Neo1*, *hepcidin,* and *Id1* mRNA transcripts in the mouse liver was normalized to the reference gene *β-actin.* The abundance of *Hepcidin, Neo1, NEO1, ID1, PSEN1 and 2* mRNA transcripts in Hep3B and HepG2 cells was also normalized to the reference gene *β-Actin*. Results are expressed as the amounts relative to that of *β-actin* (2^–ΔΔCt^). We used two different pairs of *Neo1* primers, which amply the sequences at the Neo1 Ig domains and the Neo1 intracellular domain, respectively, for analysis.

### Immunodetection

Protein extracts from transfected Hep3B cells, HepG2 cells, whole liver tissues, liver membrane preparations, as well as the concentrated FLAG-tagged Neo1 by using anti-FLAG affinity gel, were separated by using SDS-PAGE (9% or 11%) under reducing conditions. Liver membrane fractions were prepared as previously described (68). FLAG-tagged Neo1 and mutants from ∼2 mg liver extract proteins were pulled down by using anti-FLAG affinity gel (Sigma #A2220), followed by elution with 3xFLAG peptide at ∼200 μg/ml (Sigma F4799). The fNeo1 and mutants in both transfected Hep3B cells and transduced liver were probed directly by using an HRP-coupled mouse anti-FLAG M2 IgG (Sigma) and chemiluminescence. The endogenous Neo1, Tfr2, Na^+^K^+^ ATPase, and β-actin were detected by using rabbit anti-NEO1 FNIII 1 to 6 (37), purified rabbit anti-Tfr2 (69), mouse anti-Na^+^K^+^ ATPase (Santa Cruz Biotechnology; sc-21712), and mouse anti-β-actin (Sigma), and the corresponding secondary antibodies. The specificity of rabbit anti-NEO1 FNIII 1 to 6 antibody was validated by using HepG2 cells with siRNA knockdown of endogenous NEO1 (37) and the *Neo1*^*fl/fl*^*;Alb-Cre*^+^ mice (28). The specificity of rabbit anti-Tfr2 was validated by using Tfr2-deficient mice (69). All images were captured by using the c600 Western blot imaging system (Azure Biosystems, Inc). The intensities of specific bands were quantified by using the ImageJ software.

### Statistical analysis

Two-tailed student-T test was used to compare two sets of data. One-way ANOVA and Tukey’s post-test were used for multiple comparisons with Prism 9 (GraphPad). *p* < 0.05 was considered significant.

## Data availability

Raw data are available from the corresponding author on reasonable request.

## Supporting information

This article contains supporting information.

## Conflict of interest

The authors declare that they have no conflicts of interest with the contents of this article.
