# Supplementary material for: Hepcidin expression is associated with increased γ-secretase–mediated cleavage of neogenin in the liver
Source: J Biol Chem. 2024 Oct 24;300(12):107927. doi: 10.1016/j.jbc.2024.107927 (PMC11599459; doi:10.1016/j.jbc.2024.107927)
Supplement: Supplemental Tables S1-S2 and Figures S1-S8 [file mmc1.pdf]

Hepcidin expression is associated with increased γ-secretase-mediated cleavage of neogenin in the liver

Caroline A. Enns, Richard H. Zhang, Shall Jue, and An-Sheng Zhang

List of supporting information:

Table S1
Table S2
Figure S1
Figure S2
Figure S3
Figure S4
Figure S5
Figure S6
Figure S7
Figure S8

Supplemental Table S1. Primers used for generation of pCMV6-fNeo1-ECD/TMD, fNeo1-ICD, and fNeo1$^{\Delta Ig}$ constructs

**Mutagenesis primers:**	
fNeo1-ECD/TMD-Forward:	5'-CCTCTCACCAGAAGAAGAAACGAACGCGTACGCGGCCGCTCGAGC-3'
fNeo1-ECD/TMD-Reverse:	5'-GCTCGAGCGGCCGCGTACGCGTTCGTTTCTTCTTCTGGTGAGAGG-3
fNeo1-ICD-Forward:	5'-GCCGCCGCGATCGCCATGTGTACCCGGCGCACCACC-3'
fNeo1-ICD-Reverse:	5'-GGTGGTGCGCCGGGTACACATGGCGATCGCGGCGGC-3
fNeo1$^{\Delta Ig}$-Forward:	5'-GCTCGGACGCCCGGCGCCTTCAGCTCCTCGAG-3'
fNeo1$^{\Delta Ig}$-Reverse:	5'-CTCGAGGAGCTGAAGGCGCCGGGCGTCCGAGC-3'

Supplemental Table S2. List of primers used for qRT-PCR analysis

Gene	Forward primer	Reverse primer
**Mouse primers**		
β-actin	5'-CTGCCTGACGGCCAGGT-3'	5'-TGGATGCCACAGGATTCCAT-3'
Hepcidin	5'-CACCAACTTCCCCATCTGCATCTT-3'	5'-GAGGGGCTGCAGGGGTGTAGAG-3'
Id1	5'-ACCCTGAACGGCGAGATCA-3'	5'-TCGTCGGCTGGAACACATG-3'
Neo1 (1)	5'-AGATGATCGACGCCAGCTACTC-3'	5'-TCGTCAGGCTTATTGTGTTTGG-3'
Neo1 (2)	5'-CGAGCTGCGTGCAAATCA-3'	5'-ATCCATAGGTCTGGAGGCTTCAC-3'
**Human primers**		
β-Actin	5'-AGGTCATCACCATTGGCAATG-3'	5'-GTCACACTTCATGATGGAGTTGAAG-3'
HEPCIDIN	5'-GGCTCTGTTTTCCCACAACAG-3'	5'-TCCTTCGCCTCTGGAACATGG-3'
NEO1	5'-AGGTCGAAGATCAAGTACATGGAGTAT-3'	5'-CCTCTTTACTCACAACAGTCACATCCT-3'
PSEN1	5'-GACGACCCCAGGGTAACTC-3'	5'-ACTGACTTAATGGTAGCCACGA-3'
PSEN2	5'-GCTGTTTGTGCCTGTCACTCTG-3'	5'-TGTGTCCTCAGTGAATGGCGTG-3'
ID1	5'-ACGATCGCATCTTGTGTCGCT-3'	5'-AGACCCACAGAGCACGAATT-3'

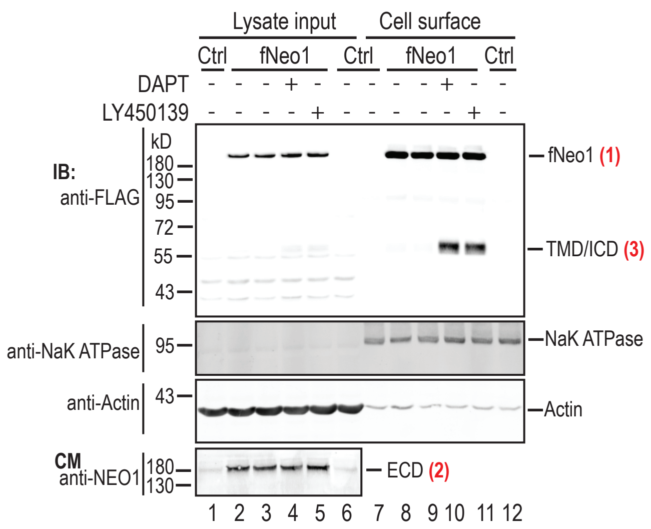

Supplemental Figure S1. *Inhibition of γ-secretase increases the α-secretase-cleaved fNeo1-TMD/ICD on the cell surface*. Transiently transfected Hep3B cells with pCMV6-fNeo1 construct were incubated in the presence of 10 µM DAPT or 10 µM LY450139 in Opti-MEM/1% FCS for about 7 hr. Cell surface proteins were biotinylated at 4˚C, followed by pull-down of the biotinylated proteins using streptavidin agarose beads. The eluted cell surface proteins, ~10% of input lysate, and a fraction of concentrated CM (~600 µl) were subjected to SDS-PAGE (11%) and immunodetection by using anti-FLAG, NEO1, Na+K+ ATPase, and β-actin antibodies. Na+K+ ATPase, a cell surface protein, served as a positive control and β-actin, a cytosolic protein, served as negative control to assure that the cells were not permeable. Experiments were repeated at least three times with consistent results.

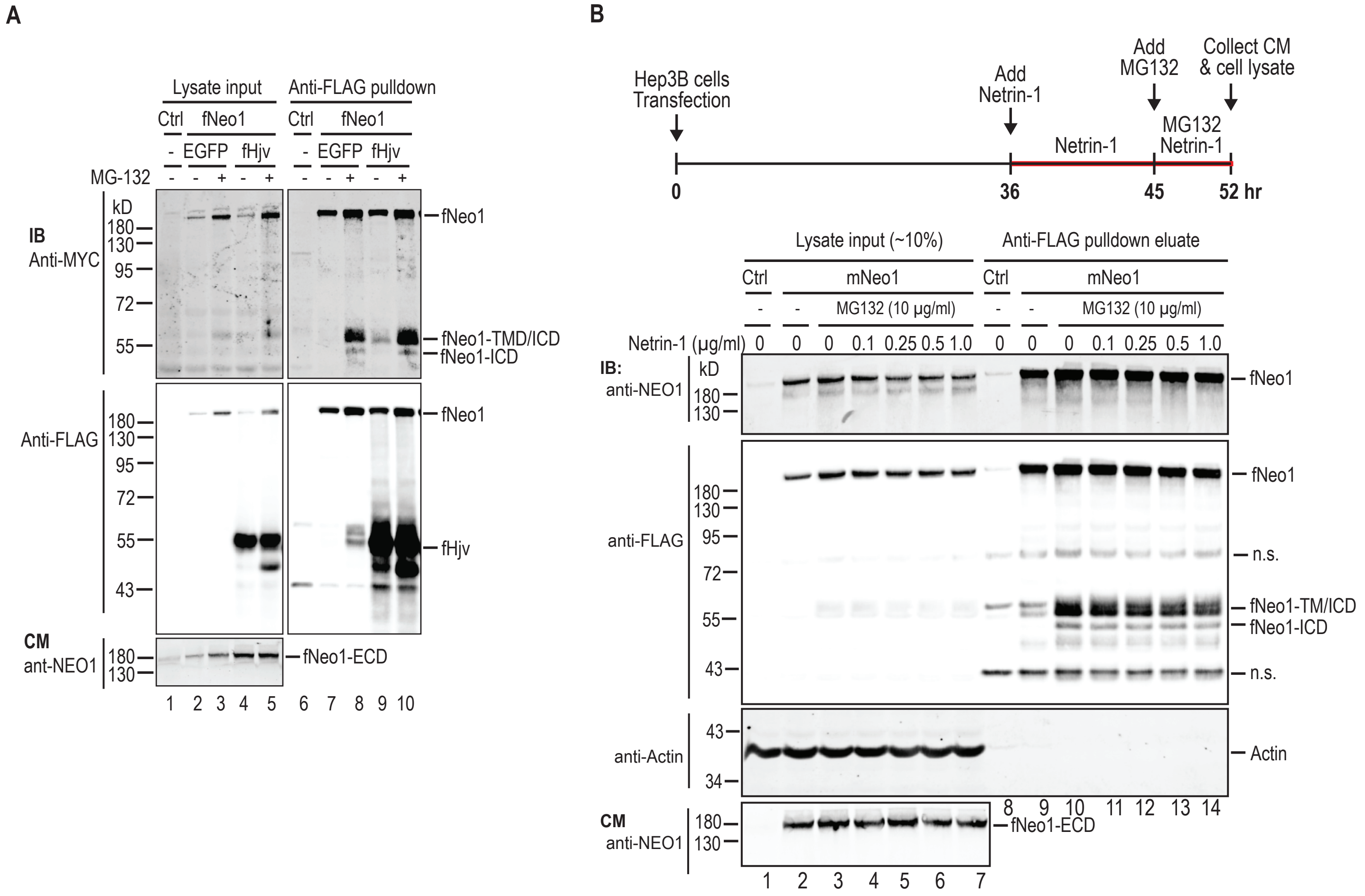

Supplemental Figure S2. **The γ-secretase cleavage of fNeo1 is not affected by either Hjv or Netrin-1 in Hep3B cells. A)** Co-expression with fHjv does not affect γ-secretase cleavage of fNeo1. Hep3B cells were transiently transfected with pCMV6-fNeo1 and pCMV9-fHjv or pEGFP-N1 (EGFP) constructs. At about 40 hr after transfection, cells were treated with 10 µM MG-132 or vehicle for about 7 hr. About 90% of cell lysate was subjected to pull-down using anti-FLAG affinity gel (Sigma #A2220) as described in the legend to **Figure 3B**. The eluted proteins, ~10% of input lysate, and a fraction of concentrated CM (~600 µl) were subjected to SDS-PAGE (11%) and immunodetection (IB) by using anti-MYC, FLAG, and NEO1 antibodies for cell-associated fNeo1, fHjv, and the shed Neo1 in CM, respectively. **B)** Incubation with Netrin-1 does not affect γ-secretase cleavage of fNeo1. The experiments were performed as described in the legends to **Figure 3B** except that 10 µM MG-132 and different concentrations of Netrin-1 were added at about 45 hr post-transfection. All experiments were repeated at least three times with consistent results.

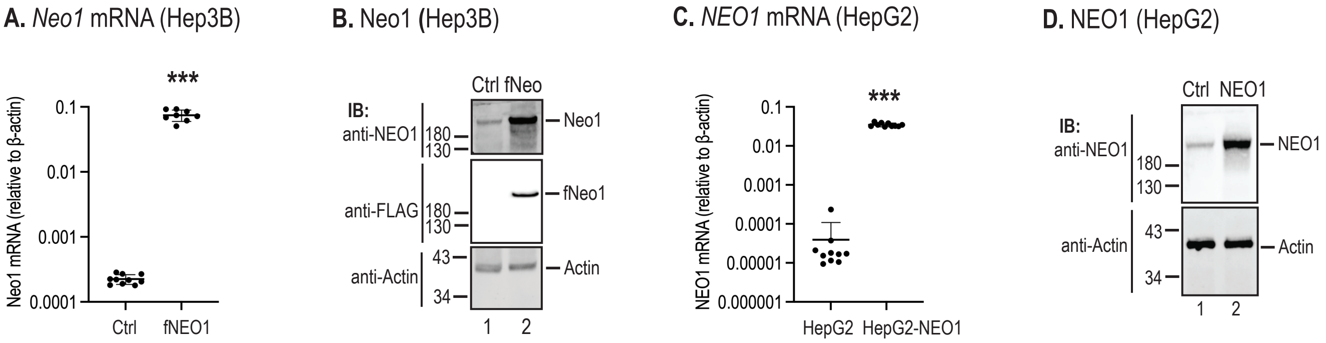

Supplemental Figure S3. **Hep3B and HepG2 cells endogenously express low levels of NEO1. A)** qRT-PCR analysis of NEO1/Neo1 mRNA in Hep3B cells with a transient transfection with empty vector (Ctrl) or pCMV9-fNeo1. Analysis was performed at about three days post-transfection. Two-tailed student-T test was used to analyze the data. **B)** Western blot analysis of the transfected fNeo1 by using anti-NEO1 and FLAG antibodies, as well as the endogenous NEO1 in empty vector-transfected Hep3B cells by using an anti-NEO1 antibody. **C)** qRT-PCR analysis of NEO1 mRNA in HepG2-Ctrl and HepG2-NEO1 cells. Two-tailed student-T test was used to analyze the data. **D)** Western blot analysis of the endogenous and transfected NEO1 in HepG2-Ctrl and HepG2-NEO1 cells.

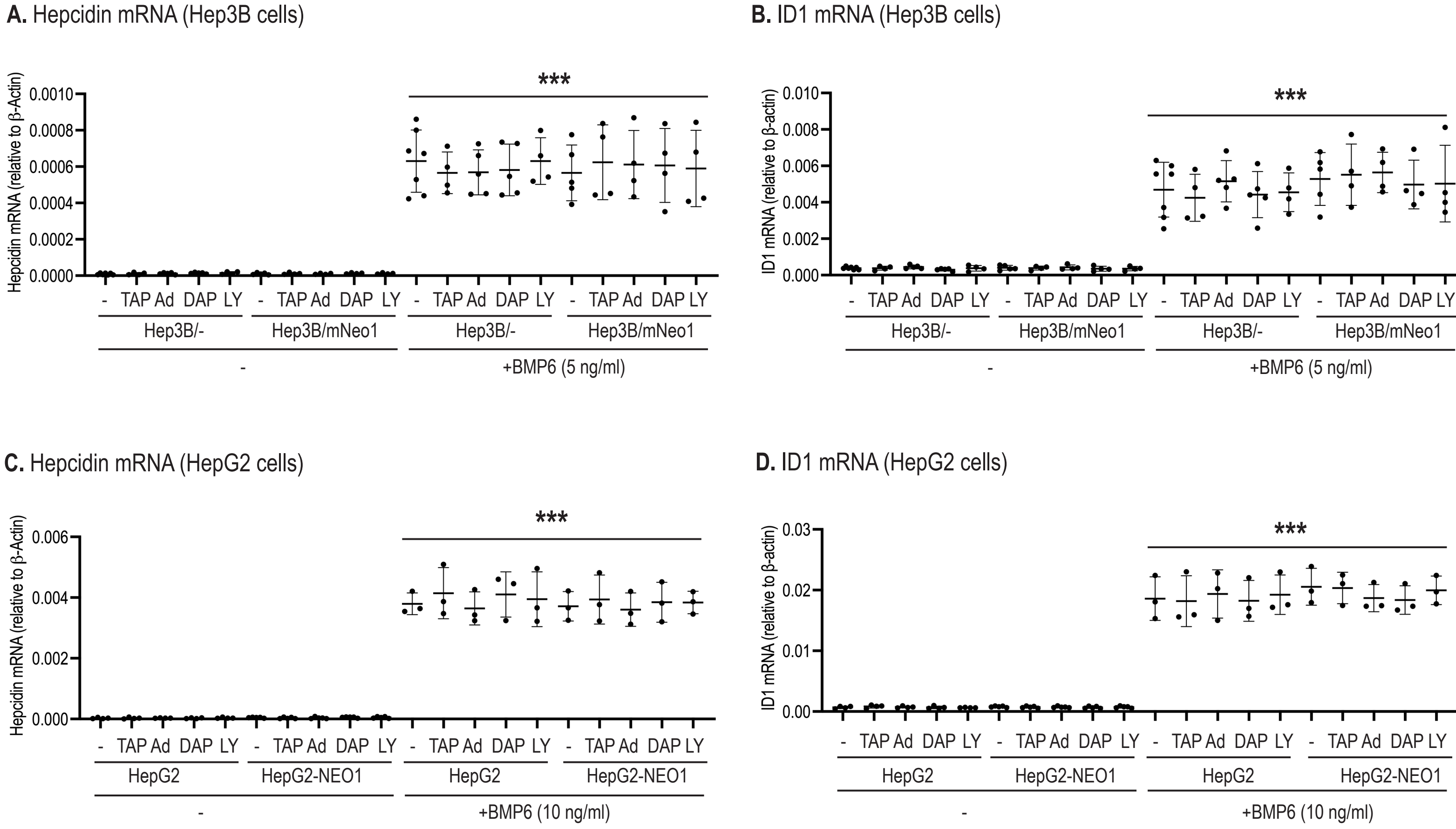

Supplemental Figure S4. **Inhibition of neither α- nor γ-secretase alters hepcidin expression in Hep3B and HepG2 cells. A-B)** qRT-PCR analysis of hepcidin and *ID1* mRNA in Hep3B cells with a transient transfection of empty vector (-) or pCMV9-fNeo1. At about 36 hr post-transfection, cells were first incubated with Opti-MEM/1% FBS for 12 hr for serum starvation. Cells were then changed to fresh Opti-MEM/1% FBS with TAPI-1 (TAP; 25 μM), Aderbasib (Ad; 25 μM), DAPT (DAP; 10 μM), LY450139 (LY; 10 μM), or vehicle (-) and incubated in the presence of 0 and 5 ng/ml BMP6 for additional 6 hr, followed by qRT-PCR analysis. Results are derived from at least four experiments. One-way ANOVA and Tukey's post-test were used to analyzed the data relative to the empty vector-transfeced controls with no inhibitor and BMP6. **C-D)** qRT-PCR analysis of hepcidin and *ID1* mRNA in HepG2-Ctrl and HepG2-NEO1 cells. The serum starving and treatment with inhibitors and BMP6 were performed as described above for Hep3B cells. Results are derived from three experiments. One-way ANOVA and Tukey's post-test were used to analyze the data relative to HepG2-Ctrl (HepG2) cells with no inhibitor and BMP6. ***, $P<0.001$.

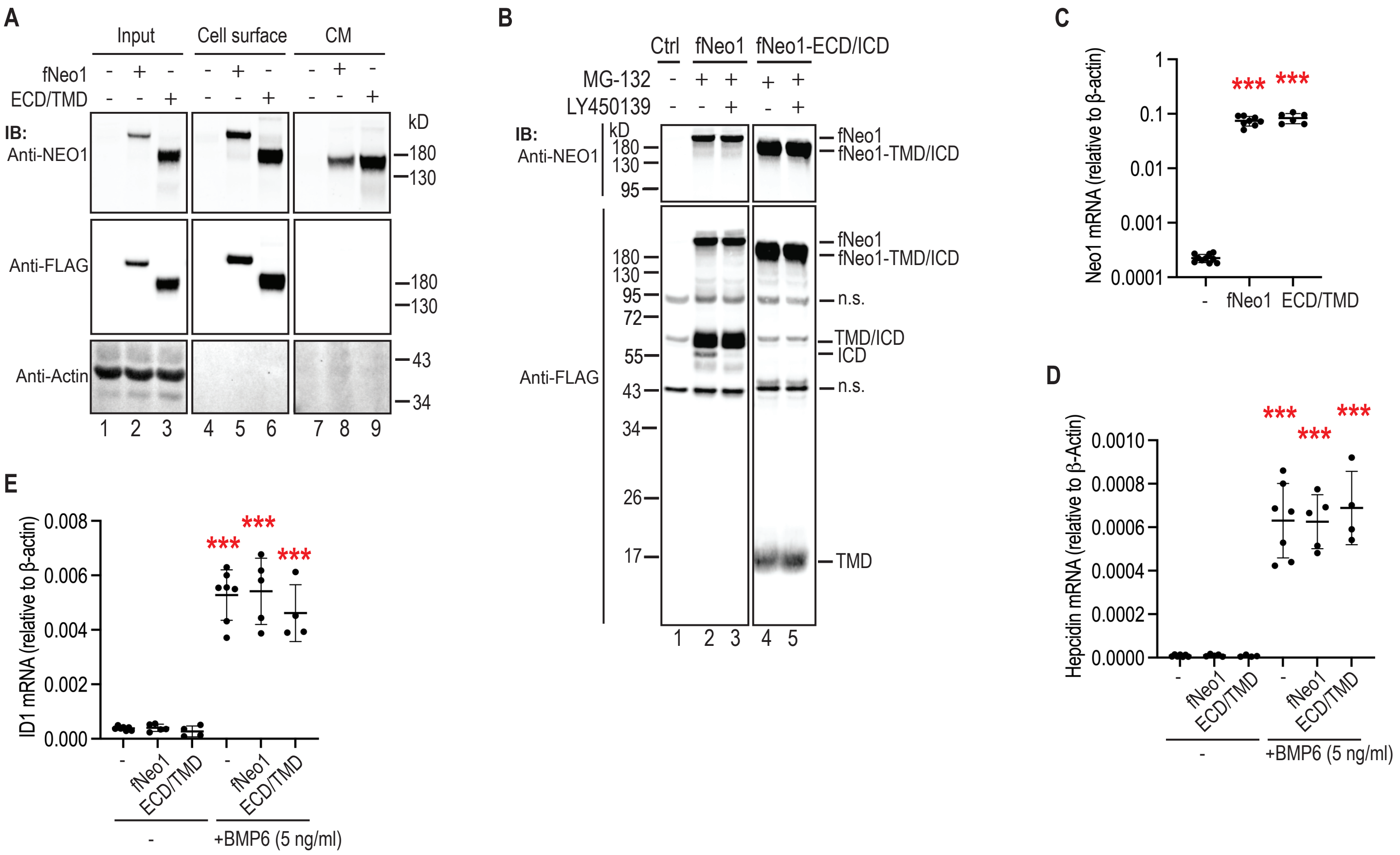

Supplemental Figure S5. *Increased expression of neither full-length Neo1 nor truncated Neo1 in Hep3B cells induces hepcidin expression.* **A)** Lack of the Neo1 intracellular domain does not affect its cell surface localization and the ectodomain shedding. Transfected cells were subjected to biotinylation of cell surface proteins and immunodetection. Each panel was cropped from the same image. Experiments were repeated three times with consistent results. **B)** Incubation with γ-secretase inhibitor, LY450139, does not alter the levels of α-secretase-cleaved Neo1 transmembrane domain (TMD) in fNeo1-ECD/TMD-expressing cells. Each panel was cropped from the same image. Experiments were performed as described in the legend to **Figure 3B** and repeated three times with consistent results. **C-E)** qRT-PCR analysis of *Neo1*, hepcidin, and *ID1* mRNA in Hep3B cells with a transient transfection with empty vector (-), pCMV9-fNeo1, or fNeo1-ECD/TMD after incubation with and without BMP6 (5 ng/ml) for 6 hr. Results are derived from at least four experiments and are expressed as the amount relative to that of β-actin for each sample. All data shown are means ± SD. One-way ANOVA and Tukey's post-test were used to analyze the data relative to the empty vector-transfected control group with no BMP6. ***, $P<0.001$.

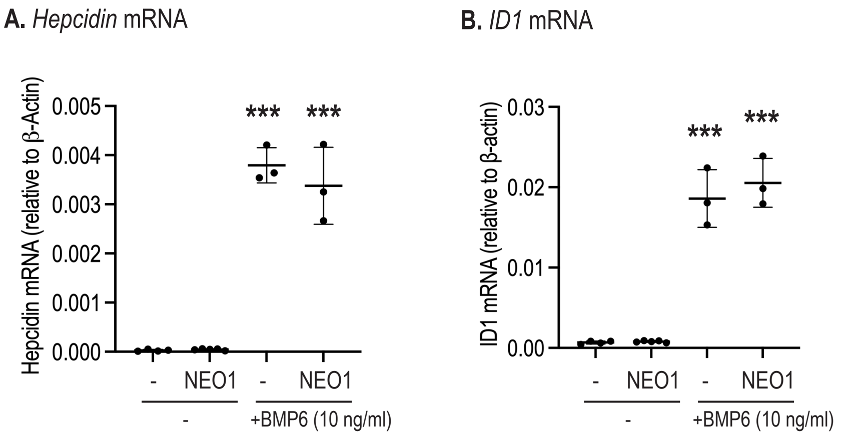

Supplemental Figure S6. **Increased expression of full-length NEO1 in HepG2 cells does not induce hepcidin expression.** qRT-PCR analysis of hepcidin (**A**) and ID1 mRNA (**B**) in HepG2-Ctrl and HepG2-NEO1 cells. At about 36 hr after subculture, cells were first incubated with Opti-MEM/1% FBS for 12 hr for serum starvation. Cells were then changed to fresh Opti-MEM/1% FBS with or without BMP6 at 10 ng/ml and incubated for additional 6 hr, followed by qRT-PCR analysis. Results are derived from at least three experiments. All data shown are means ± SD. One-way ANOVA and Tukey's post-test were used to analyze the data relative to the empty vector-transfected control group with no BMP6**.** ***, $P<0.001$.

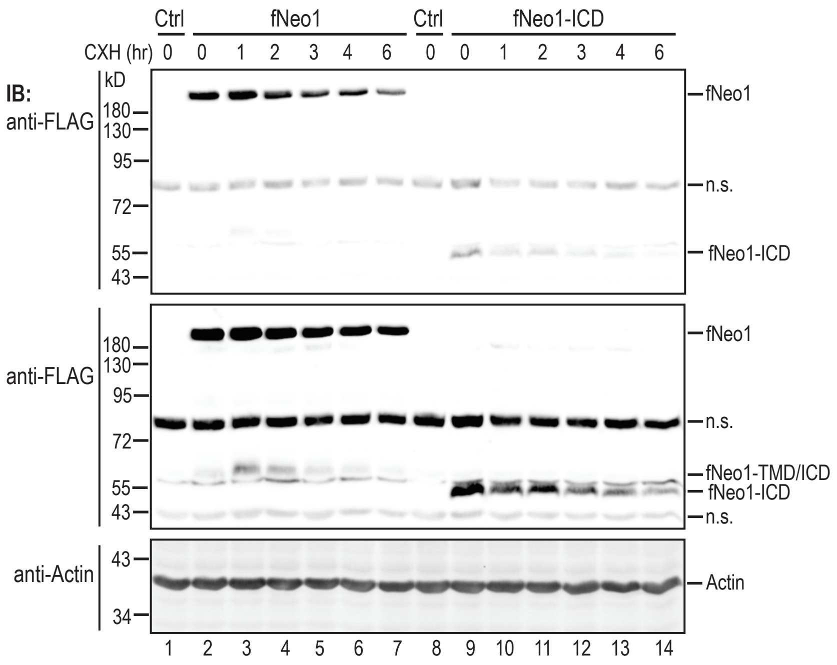

Supplemental Figure S7. *Comparison of the degradation rates of fNeo1 and fNeo1-ICD in Hep3B cells.* Transfected Hep3B cells were incubated in the complete medium with 100 μg/ml of cycloheximide (CHX) to block protein synthesis. After 0, 1, 2, 4, and 6 hr of incubation, cell lysate was prepared and subjected to SDS-PAGE, followed by immunodetection using anti-FLAG and β-actin antibodies. Experiments were repeated three times with consistent results.

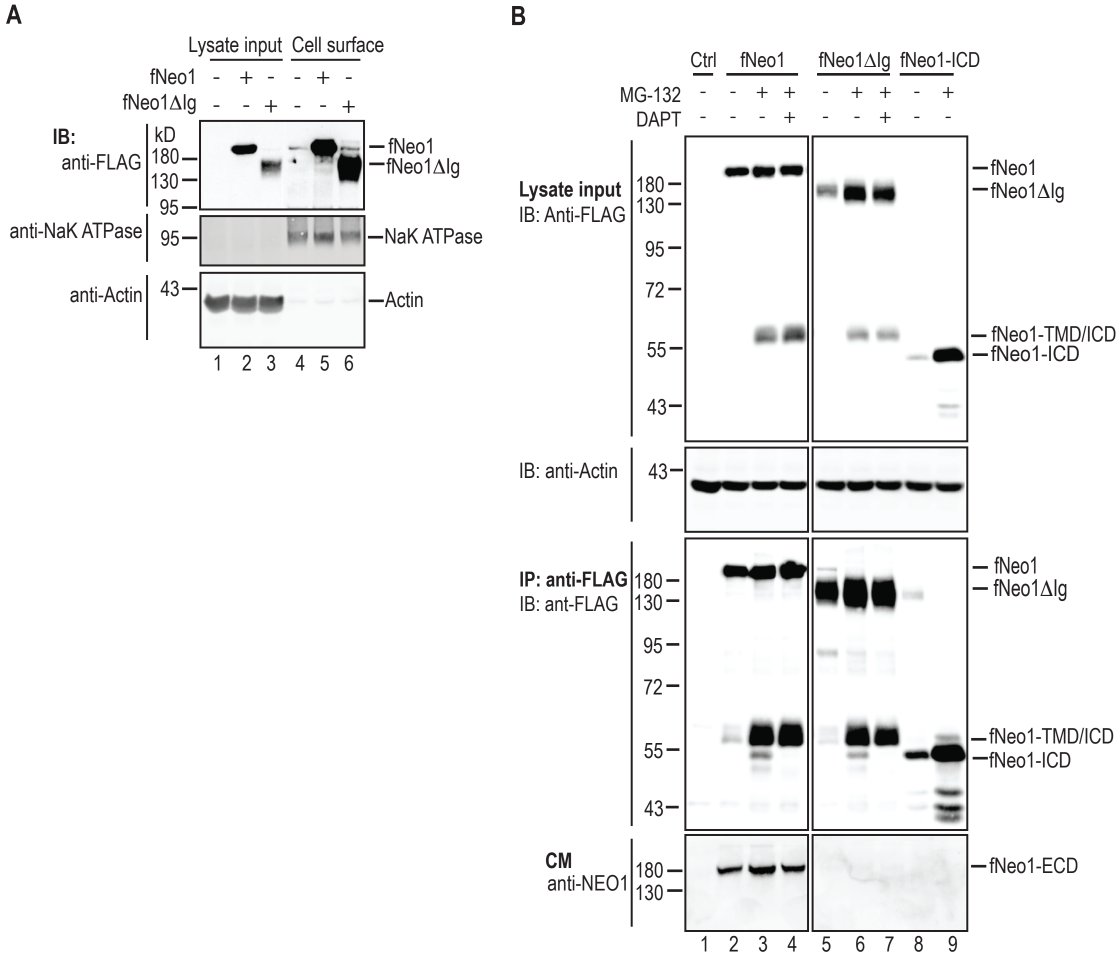

Supplemental Figure S8. *Deletion of the immunoglobulin (Ig)-like domains does not affect Neo1 cell surface localization and γ-secretase cleavage*. **A)** Lack of the Ig domains does not affect Neo1 cell surface localization in Hep3B cells. Hep3B cells were transiently transfected with pCMV6-fNeo1 or fNeo1ΔIg construct. At about 48 hr after transfection, cell surface proteins were biotinylated. The eluted cell surface proteins and ~10% of input lysate was subjected to SDS-PAGE (11%) and immunodetection (IB) by using anti-FLAG, Na+K+ ATPase, and β-actin antibodies. **B)** Deletion of the Ig domains does not affect γ-secretase cleavage of fNeo1. Hep3B cells were transiently transfected with pCMV6-fNeo1 or fNeo1ΔIg construct. At about 40 hr after transfection, cells were treated with 10 μM MG-132 and 10 μM DAPT in Opti-MEM/1% FCS for about 7 hr. About 90% of cell lysate was subjected to pull-down using anti-FLAG affinity gel (Sigma #A2220), followed by elution using the 3xFLAG peptide at ~200 μg/ml (Sigma F4799). The eluted proteins, ~10% of input lysate, and a fraction of concentrated CM (~600 μl) were subjected to SDS-PAGE (11%) and immunodetection (IB) by using anti-FLAG, NEO1, and β-actin antibodies. Each panel was cropped from the same image. All experiments were repeated three times with consistent results.
